# Analysis of the external signals driving the transcriptional regulation of the main genes involved in denitrification in *Haloferax mediterranei*

**DOI:** 10.3389/fmicb.2023.1109550

**Published:** 2023-03-16

**Authors:** Jose María Miralles-Robledillo, Rosa María Martínez-Espinosa, Carmen Pire

**Affiliations:** ^1^Biochemistry and Molecular Biology Division, Agrochemistry and Biochemistry Department, Faculty of Sciences, University of Alicante, Alicante, Spain; ^2^Multidisciplinary Institute for Environmental Studies “Ramón Margalef”, University of Alicante, Alicante, Spain

**Keywords:** haloarchaea, *Haloferax mediterranei*, denitrification, transcriptional regulation, promoter activity

## Abstract

*Haloferax mediterranei* is the model microorganism for the study of the nitrogen cycle in haloarchaea. This archaeon not only assimilate *N*-species such as nitrate, nitrite, or ammonia, but also it can perform denitrification under low oxygen conditions, using nitrate or nitrite as alternative electron acceptors. However, the information currently available on the regulation of this alternative respiration in this kind of microorganism is scarce. Therefore, in this research, the study of haloarchaeal denitrification using *H. mediterranei* has been addressed by analyzing the promoter regions of the four main genes of denitrification (*narGH*, *nirK*, *nor,* and *nosZ*) through bioinformatics, reporter gene assays under oxic and anoxic conditions and by site-directed mutagenesis of the promoter regions. The results have shown that these four promoter regions share a common semi-palindromic motif that plays a role in the control of the expression levels of *nor* and *nosZ* (and probably *nirK*) genes. Regarding the regulation of the genes under study, it has been concluded that *nirK*, *nor,* and *nosZ* genes share some expression patterns, and therefore their transcription could be under the control of the same regulator whereas *nar operon* expression displays differences, such as the activation by dimethyl sulfoxide with respect to the expression in the absence of an electron acceptor, which is almost null under anoxic conditions. Finally, the study with different electron acceptors demonstrated that this haloarchaea does not need complete anoxia to perform denitrification. Oxygen concentrations around 100 μM trigger the activation of the four promoters. However, a low oxygen concentration *per se* is not a strong signal to activate the promoters of the main genes involved in this pathway; high activation also requires the presence of nitrate or nitrite as final electron acceptors.

## 1. Introduction

Denitrification is one of the main pathways of the nitrogen cycle. This process encompasses a series of redox reactions in which nitrate (NO_3_^−^) can be reduced sequentially to nitrite (NO_2_^−^) by the respiratory nitrate reductase (encoded by the *narGH* genes), nitric oxide (NO) by the respiratory nitrite reductase (encoded by the *nirK* gene), nitrous oxide (N_2_O) by the nitric oxide reductase (encoded by the *nor* gene), and finally, dinitrogen (N_2_) by the nitrous oxide reductase (encoded by the *nosZ* gene) (Richardson et al., 2009). This process shows a close relationship with climate change, especially due to two of their intermediates that participate directly or indirectly in ozone layer depletion: NO and N_2_O (Ehhalt et al., 2001; Richardson et al., 2009; Cavigelli et al., 2012; Myhre et al., 2013). NO is considered a precursor of greenhouse gasses, whereas N_2_O shows a potent greenhouse effect, being 300 times as potent as carbon dioxide (CO_2_) at heating the atmosphere (Ehhalt et al., 2001; Cavigelli et al., 2012; Myhre et al., 2013; Masson-Delmotte et al., 2019). Due to this reason, worldwide efforts in mitigating their release into the atmosphere have caused an increase in the number of publications that correlate denitrification with climate change (Masson-Delmotte et al., 2019; Miralles-Robledillo et al., 2021).

N_2_O emissions mostly come from agricultural practices due to soil microbial metabolism in fertilized soils (Cavigelli et al., 2012; Zhang et al., 2016; Feng et al., 2018; Muntean et al., 2019; Olivier and Peters, 2019). Therefore, many studies about denitrification focus on these systems aiming to improve the use of *N*-species as fertilizers (Shcherbak et al., 2014; Wang et al., 2018). Nevertheless, other less studied and underestimated systems in terms of N_2_O emissions are saline and hypersaline environments such as submarine salt domes, salted lagoons, or salt marshes (Torregrosa-Crespo et al., 2018). The exact extent of these ecosystems on our planet is not known, although it has been described that they are increasing due to climate change and anthropogenic activities contributing to the expansion of arid and semiarid ecosystems (Delgado-Baquerizo et al., 2013; Medhaug et al., 2017; Torregrosa-Crespo et al., 2018; Schuler et al., 2019). Moreover, intakes of nitrate, nitrite, and ammonium in these areas are rising due to anthropogenic activities (Firestone et al., 1980; Conrad, 1996; Kool et al., 2011; Martínez-Espinosa et al., 2011).

Nowadays, studies carried out in these saline/hypersaline environments have been mainly related to the ecology of living beings that can tolerate the presence of high salt concentrations as well as to the characterization of microbial population interactions. It has been reported that in hypersaline environments the microbial population which is most prevalent is the haloarchaea, the *Haloferacaceae* family being the best well-studied (Oren, 2002). Some important representatives of this family are *Haloferax, Haloarcula,* and *Natronomonas* genera due to their possible biotechnological applications and their important role in the biogeochemical cycles of these ecosystems (Bonete et al., 2008; Giani et al., 2019; Miralles-Robledillo et al., 2021). In this context, the *Haloferax* genus is the best characterized from the biochemical point of view and it has been chosen as a model organism in many studies in order to decipher how N-cycle works in hypersaline environments (Martínez-Espinosa et al., 2007; Torregrosa-Crespo et al., 2016).

*Haloferax mediterranei* is the model archaeon for dissimilatory nitrogen metabolism studies due to its metabolic versatility. Not only can it assimilate nitrate, nitrite, and ammonia, but also shows the capacity for denitrification, being capable of reducing completely nitrate to dinitrogen using this route as alternative respiration to standard aerobic respiration (Esclapez et al., 2014; Oren, 2017; Torregrosa-Crespo et al., 2019). This adaptation is advantageous for the cells since saline systems show low oxygen solubility and therefore oxygen concentration is usually lower than in mesophilic environments (Oren, 2016). Over the years, haloarchaeal denitrification has been explored from different points of view: efforts have focused on the purification of the main enzymes and more recently on bioinformatic analyzes and the physiological characterization of denitrification (Lledó et al., 2004; Esclapez et al., 2013; Torregrosa-Crespo et al., 2017, 2019). However, unlike in bacteria, little is known about the genetic regulation of denitrification in this type of microorganism, which represents a major knowledge gap about this pathway (Hattori et al., 2016; Torregrosa-Crespo et al., 2020a; Koyanagi et al., 2021). Furthermore, there are no homologous genes in haloarchaea for bacterial regulators related to denitrification such as FnrP, NNR, or NarR, which makes it more difficult to identify the genetic network controlling this alternative respiration (Spiro, 2016).

Torregrosa-Crespo et al. (2020a) studied gas emissions due to the growth of *H. mediterranei* under anaerobic growth in the presence of nitrate and nitrite. In addition, they also performed a transcriptomic analysis of the four main denitrification genes (*narGH*, *nirK*, *nor,* and *nosZ*) under denitrification conditions (presence of nitrate and anaerobiosis) (Torregrosa-Crespo et al., 2020a). Nevertheless, to understand haloarchaeal denitrification in depth, it is essential to explore different conditions that could provide information about denitrification activation as well as about the nature of the signals that can modulate this alternative respiration in hypersaline environments. Thus, in this study *H. mediterranei* anaerobic growth and denitrification promoter activation have been analyzed under the presence of different final alternative electron acceptors (NO_3_^−^, NO_2_^−,^ and DMSO) and without them.

This work has delved into the regulation of denitrification in *H. mediterranei* through bioinformatic and experimental analysis of the promoter regions of the genes encoding the four main enzymes of denitrification. Common regulatory sequences have been found in their promoter regions and promoter activities have been tested under different growth conditions aiming to complete the regulatory model for this respiratory pathway in haloarchaea.

## 2. Materials and methods

### 2.1. Reporter gene assay: Promoter cloning and β-galactosidase enzymatic assay

Reporter gene assays were performed using the *β*-galactosidase (*bgaH*) gene from *Haloferax lucentense* as a reporter gene cloned in an expression plasmid. The plasmid used (pVA513) was a modified version of the plasmid pMDS132 with an extra *Nco*I restriction site at the start ATG of the *bgaH gene* (this plasmid was kindly provided by Dr. Mike Dyall-Smith, University of Melbourne, Australia) (Holmes and Dyall-Smith, 2000). The vector map is displayed in Supplementary Figure S1A.

Promoter regions of the studied genes (Table 1; Supplementary Figures S2, S3) were amplified by PCR from *H. mediterranei* R4 ATCC 33500 genomic DNA (genome consists of one chromosome of 2,948,887 bp and three megaplasmids, pHME132 of 131.975 bp, pHME322 of 321.907 bp, and pHME505 of 504,704 bp) (Rodriguez-Valera et al., 1980; DasSarma et al., 2019). For these amplifications, all the spaces between two coding regions were considered promoter regions ensuring that the possible TATA and BRE boxes are inside this amplified region. Restriction sites for *Nco*I and *Hind*III enzymes were added to the primers used for subsequent cloning into the corresponding vector. PCR products were purified using the GFX PCR DNA and Gel Band Purification Kit from Cityva and cloned into pJET1.2 linearized plasmid using CloneJET PCR Cloning Kit (Thermo Scientific) (Vector map is shown in Supplementary Figure S1B). *Escherichia coli* JM110 was transformed by the heat-shock method with pJET1.2 plasmid containing promoter regions to produce a high amount of unmethylated plasmid and facilitate restriction (Asif et al., 2017). The pJET1.2 constructions were linearized by the *Nco*I and *Hind*III restriction enzymes, and the inserts were purified with the E.Z.N.A.^®^ Plasmid DNA Mini Kit II (Omega Bio-tek) and ligated in the pVA513 plasmid using T4 DNA ligase (Thermo Scientific). Escherichia coli JM110 were transformed with pVA513 constructs by the heat-shock method (Asif et al., 2017). The plasmid was isolated from *E. coli* JM110 with the E.Z.N.A.^®^ Plasmid DNA Mini Kit II and transformed into *H. mediterranei* R4 ATCC 33500 using the standard PEG-mediated transformation of haloarchaea (Dyall-Smith, 2009). The correct vector construction was verified by sequencing the plasmid isolated from *H. mediterranei*. Sequencing was carried out by the Research Support Services of the University of Alicante using the Sanger method. PCR product purification, plasmid purification, and ligation were carried out following manufacturer instructions. Transformed strains of *H. mediterranei* R4 ATCC 33500 and its respective genotypes are shown in Table 2.

**Table 1:** List of primers and genes whose promoter regions were amplified.

Gene (Location)	Sequence	Position	Amplicon size (bp)
*narGH* (pHME322)	GTCGCCTCCAAGCTTTCTTCCGC	20220–20198	420
	AAGATGGCCATGGTCTCTCGCCTCAT	19801–19826	
*nirK* (chromosome)	CGGGCAACAAGCTTCGGTCACG	151234–151255	267
	ACGTCCGTCCCATGGTTGTTGATAGCAT	151500–151473	
*nor* (chromosome)	GGGAGCTCATCAAAGCTTGAAGTAACGG	145162–154189	159
	TTCGCCATGGTCTTACGCTTGAGTTCCAT	145320–145292	
*nosZ* (pHME322)	CGTTGACTAAGCTTCCGGGTGAGCAC	4829–4804	501
	CGTTCCCCATGGTATGTTTCCCTGCCAT	4329–4359	

**Table 2:** Strains of *H. mediterranei* used in reporter gene assays.

Strain	Promoter under study	Genotype
pVA513 + *narp*	*nar* promoter (*narp*)	*narp*::bgaH + nov*
pVA513 + *nirp*	*nir* promoter (*nirp*)	*nirp*::bgaH + nov*
pVA513 + *norp*	*nor* promoter (*norp*)	*norp*::bgaH + nov*
pVA513 + *nosp*	*nos* promoter (*nosp*)	*nosp*::bgaH + nov*

^*^nov: novobiocin resistance.

Protein extracts from these cultures were prepared as follows for β-galactosidase activity measurement. An aliquot of culture was extracted and centrifuged (13,000 g, 4°C, 5 min). The volume of the aliquot was taken according to the optical density value at 600 nm (OD600 nm) of the cultures, ranging from 15 mL for lower cell density values to 5 mL for higher values. Pellet was weighed and resuspended in bgaH buffer (2.5 M NaCL, 50 mM Tris–HCl, 10 μM MnCL_2_, 0.1% (v/v) β-mercaptoethanol pH 7.2) at 30% (w/v). This mixture was sonicated and centrifuged again (13,000 g, 4°C, 10 min) to obtain the protein extract (supernatant). The protein concentration of extracts was measured by Bradford assay to calculate specific activity (Bradford, 1976). The β-galactosidase activity was assayed as described previously (Dyall-Smith, 2009): 25 μL to 400 μL of protein extracts were diluted to a final volume of 950 μL using bgaH buffer and heated at 40°C for 3 min. The volume of the extract was adjusted based on β-galactosidase activity. After this time, 50 μL ortho-nitro-phenol β-D-galactoside (ONPG) was added at a final concentration of 0.4 μg/μL to the reaction. Finally, the release of o-nitrophenol (ONP) from ONPG was monitored for 5 min following the increase in absorbance at 405 nm. Measurements were done in triplicate. One unit of enzyme activity was defined as the amount of enzyme necessary to form 1 μmol of ONP per minute under the assay conditions. Specific activity (U/mg of protein) was calculated from the initial slope of the absorbance vs. time plot, using the following equation (Ɛ: molar extinction coefficient of ONP):


$$\frac{U}{\mathrm{mg}}=\;\frac{\frac{\Delta A_{405}}{\min}.\mathrm{Reaction}\;\mathrm{vol}.\;\left(L\right)\cdot 10^{6}}{\varepsilon \;ONP\left(\;M^{-1}\cdot cm^{-1}\right)\cdot \mathrm{Extract}\;\mathrm{vol}.\;\left(\mathrm{mL}\right).\;\;\mathrm{Protein}\;\mathrm{conc}.\;\left(\frac{\mathrm{mg}}{\mathrm{mL}}\right)}$$

### 2.2. Strain used, culture media, and cell growth calculations

*Haloferax mediterranei* R4 transformed strains were grown in defined media: 27.75 mM glucose, 1 mM Pi (K_2_HPO_4_/KH_2_PO_4_), 15 mM NH_4_CL, 0.03 mM FeCL_3_, 2.67 M NaCL, 0.16 M MgSO_4_·7H_2_O, 0.133 M MgCL_2_·6H_2_O, 53.33 mM KCL, 1.6 mM NaHCO_3_, 4.53 mM NaBr 6.6 mM CaCL_2_, 253.8 mM novobiocin and buffered with 0.1 M (3-(N-morpholino) propanesulfonic acid (MOPS) (Rodriguez-Valera et al., 1980; DasSarma et al., 2019). Media were supplemented with different electrons acceptor depending on the assay: 10 mM KNO_3_, 2 mM NaNO_2_, and 15 mM dimethyl sulfoxide (DMSO). These concentrations were selected based on previous studies (Oren and Trüper, 1990; Torregrosa-Crespo et al., 2019, 2020b). Cultures were set up at pH 7.3 using a NaOH solution and 42°C for oxic and anoxic conditions. Cellular growth was monitored spectrophotometrically by measuring the OD_600 nm_. Growth-specific velocity (μ) was calculated by obtaining the slope of the semi-log graph in the exponential growth phase during anaerobiosis. The slope value was the average value for all the growth curves under the same condition, regardless of the strain used. Cultures and measurements were done in triplicate.

### 2.3. Aerobic growth: O_2_, NO_3_^−^, NO_2_^−^ and promoter activation monitoring

Cell cultures of the different strains used in this study were grown in defined media supplemented with NO_3_^−^ and set up on Duran bottles at 42°C on a rotary shaker at 170 rpm agitation rate to ensure high oxygen exchange between media and air chamber. Air chambers of these cultures were continuously renewed when taking samples ensuring the maximum air saturation in the media during the growth. Dissolved oxygen concentration in the culture media was measured using oxygen-dependent luminescence sensor spots (OXSP5, PyroScience). Spots were affixed to the inner side of flasks and were noninvasively connected to an optical oxygen meter (FireStingGO2, Pyroscience) for measurements. Dissolved oxygen concentration and oxygen solubility calculations were based on the parameterized model proposed by Geng and Duan for saltwater and brines (Geng and Duan, 2010). Extracellular NO_3_^−^ and NO_2_^−^ concentrations were also determined in these cultures following spectrophotometric assays as previously described (Griess, 1879; Snell and Snell, 1944).

For NO_3_^−^ monitorization, 1 mL of cell culture was centrifuged at 13,000 g for 2 min. The supernatant was recovered and diluted to 1:100 with water. Subsequently, 20 μL of HCL 1 N was added to the supernatant and this mixture was incubated at room temperature for 10 min. Finally, the sample was measured spectrophotometrically by measuring the absorbance (Abs) value at 220 nm and 275 nm (this value is used to correct for interferences with organic matter). Measurements were done in triplicate. The NO_3_^−^ content is calculated using a standard curve and the following equation:

$$Abs=Abs_{220nm}-2\cdot Abs_{275nm}$$

Extracellular NO_2_^−^ was measured as follows: 1 mL of cell culture was centrifuged at 13,000 g for 2 min and the supernatant was recovered and diluted by convenience to 1:100 or 1:200 with water. After that, 950 μL of water were mixed with 50 μL of the sample. 1 mL of sulphanilamide solution (0.01 g/mL sulphanilamide in 3 N HCL) and 1 mL of N-(1-naphthyl)-ethylenediamine-dihydrochloride (NEDA) solution (0.02 mg/mL NEDA in water) were added to the mixture and incubated at room temperature for 20 min. Finally, the absorbance value at 540 nm of the sample was measured spectrophotometrically and NO_2_^−^ content was determined using a standard curve. Measurements were done in triplicate.

### 2.4. Anaerobic growth with different electron acceptors and promoter activation

Before anaerobic growth, cell cultures were grown aerobically in defined media supplemented with different alternative electron acceptors or without alternative electron acceptors other than O_2_ until the OD_600 nm_ reached 0.2. Then, the cultures were transferred to Duran glass bottles with a bromobutyl rubber closure that does not allow air exchange and keeps a small air chamber ( < 10% culture volume). Samples of these cultures were extracted by using sterile needles until the cultures reached the stationary phase of growth.

### 2.5. Regulatory motifs: Search and mutagenesis

The sequences of the promoter regions of the genes encoding the four main enzymes of denitrification (*narGH*, *nirK*, *nor,* and *nosZ*) were obtained from NCBI Genome DataBase (NCBI Genome RefSeq ID: GCF_005406325.1). These promoter regions were analyzed using the free software MEME from the online tool MEME-Suite 5.1.1^1^ to find possible common regulatory motifs (Bailey et al., 2015). In addition to this analysis, FIMO software was used to find this motif in the whole *H. mediterranei* R4 genome (NCBI Genome RefSeq ID: GCF_005406325.1), and BEDtools was used to filter the intergenic motifs found using the sub-command *closest* (Quinlan and Hall, 2010; Bailey et al., 2015). Default parameters were set for MEME and FIMO search. In these analyzes, all the spaces between two coding regions were considered promoter regions, ensuring that possible TATA and BRE boxes are inside this region.

One putative regulatory motif shared by *narp*, *nirp*, *norp,* and *nosp* was found and selected for site-directed mutagenesis. Site-directed mutagenesis was carried out using QuikChange II XL Site-Directed Mutagenesis Kit (Agilent) in pVA513 + *narp*, pVA513 + *nirp*, pVA513 + *norp*, and pVA513 + *nosp* plasmids. Table 3 displays the different mutations performed in the conserved motif of the four promoters. The effect of mutations in promoter activity was monitored by measuring β-galactosidase activity after growing the cells anaerobically for 24 h in defined media without an electron acceptor (see section 2.4).

**Table 3:** Strains of *H. mediterranei* used in reporter gene assays.

Promoter	Motif	Mutation	Strain
*nosp*	CGAACATGTTCG	**T**GAACATGTTCG	pVA513 + *nosp*MUT1
		CGAAC**GC**GTTCG	pVA513 + *nosp*MUT6 + 7
		CGAACATGTTC**A**	pVA513 + *nosp*MUT12
		CGAACATGTT**TA**	pVA513 + *nosp*MUT11 + 12
*narp*	CAACAATATTCG	**T**AACAATATTCG	pVA513 + *narp*MUT1
		**TG**ACAATATTCG	pVA513 + *narp*MUT1 + 2
*nirp*	CGAATATGTTCG	**T**GAATATGTTCG	pVA513 + *nirp*MUT1
*norp*	CGAACATGTTCG	**T**GAACATGTTCG	pVA513 + *norp*MUT1

Mutated nucleotides are marked in bold and underlined.

### 2.6. Promoter activity and cell growth comparisons: Statistical analysis

The identification of significant differences in the maximum specific activity of the different promoters among different culture conditions was performed as follows: Firstly, the variance of the different samples was compared using the Bartlett’s test (5 culture conditions and 4 promoters), and the specific activity values were represented in box and whiskers graphs (Supplementary Figure S4). Groups separated on the basis of variance, were analyzed separately by a two-way ANOVA test followed by a *post hoc* Tukey’s test to perform multiple comparisons between culture conditions and promoters within the same group. Alpha was set at 0.05 for all comparisons.

Calculations for differences in specific activity values among promoters under the same culture condition were carried out using a one-way ANOVA test followed by a Dunnett’s multiple comparison test. Alpha was set at 0.05 for all comparisons.

Statistical analysis for the site-directed mutagenesis experiments was performed using a Student’s *t*-test comparing the specific activity of the different mutations against the control to look for significant differences (value of *p* < 0.05).

Bartlett’s test and box and whiskers graph were carried out using R 4.2.2, the rest of the graphs, ANOVA test, and multiple comparison tests were performed using GraphPad Prism 8 software.

## 3. Results and discussion

### 3.1. Denitrification promoters are activated in anaerobiosis under the presence of different electron acceptors

In order to monitor the activity of denitrification promoters in anaerobiosis under the presence of different electron acceptors, the β-galactosidase specific activity was measured, as described previously, from protein extracts of *H. mediterranei* cells transformed with the four plasmids (pVA513 + *narp*, pVA513 + *nirp*, pVA513 + *norp*, and pVA513 + *nosp*), and grown in anoxic conditions in presence of alternative electron acceptor (DMSO, nitrate, and nitrite), and in the absence of electron acceptor. The variance of the maximum β-galactosidase specific activity values was analyzed, showing significant differences according to Bartlett’s test (value of *p* < 0.0001). These values were plotted in Supplementary Figure S4, displaying two different groups: Condition with high specific activity values (anaerobic cultures supplemented with nitrate or nitrite) and condition with low specific activity values (anaerobic cultures without electron acceptor, and anaerobic cultures supplemented with DMSO). These two groups presented clear differences between them and comparisons inside each group were analyzed separately (Supplementary Tables S1–S4).

**Figure 1:**
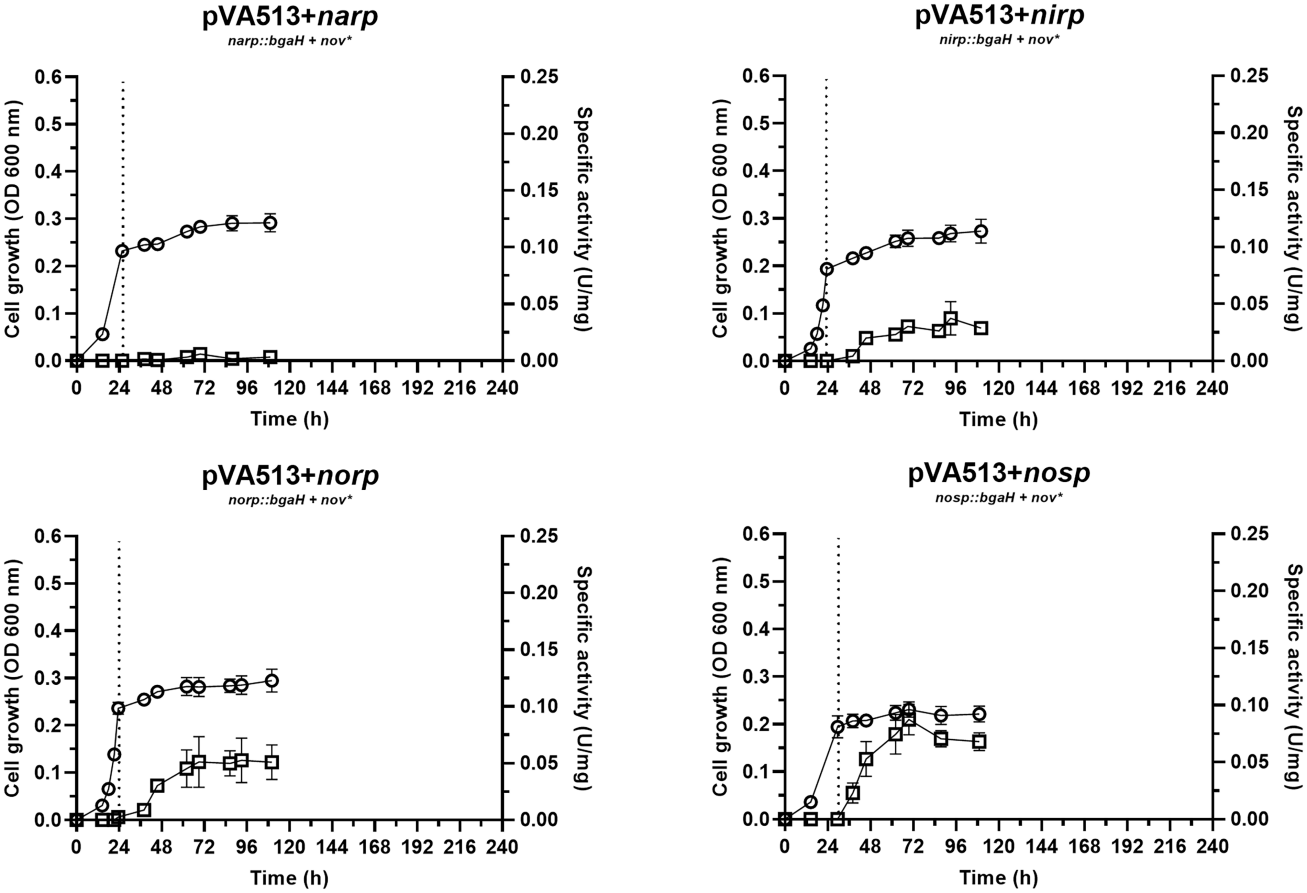
*Haloferax mediterranei* R4 anaerobic growth curves (circles) and promoter activity (squares) of the four main genes of denitrification (*narGH*, *nirK*, *nor,* and *nosZ*) without the availability of alternative electron acceptors. Promoter activity was measured using the β-galactosidase specific activity. Dotted lines represent the switch from oxic conditions to anoxic. Titles of each graph represent the specific strain used for that study. pVA513 refers to the plasmid used that carries the reporter gene (*bgaH* gene). *narp* (nitrate reductase promoter), *nirp* (nitrite reductase promoter), *norp* (nitric oxide reductase promoter), and *nosp* (nitrous oxide reductase promoter) refer to the promoter cloned in the plasmid. *Nov:* novobiocin resistance.

The condition without an electron acceptor was analyzed under anoxic conditions to explore if the sole absence of oxygen is enough for the activation of denitrification promoters. As expected, *H. mediterranei* shows almost no growth under this condition (Figure 1). Nonetheless, low specific activity has been detected for *narp, nirp*, *norp*, and *nosp.,* compared with the cultures supplemented with nitrate or nitrite (see below). Furthermore, the *nar* promoter seems to be the least responsive to anaerobiosis, showing almost no activity at 45 h compared with the rest of the promoters (Figure 1; Supplementary Tables S5, S6). These results indicate that there is probably a transcriptional regulator that triggers the promoter activation of *narp, nirp*, *norp,* and *nosp* under micro-oxic conditions, but an inducing molecule may be necessary to produce high activation. In the haloarchaeon *Haloferax volcanii*, this regulator has been proposed to be the NarO transcriptional regulator found upstream of the *nar* operon (Hattori et al., 2016). However, neither the gene encoding this regulator nor homologs have been found in the *H. mediterranei* genome.

**Figure 2:**
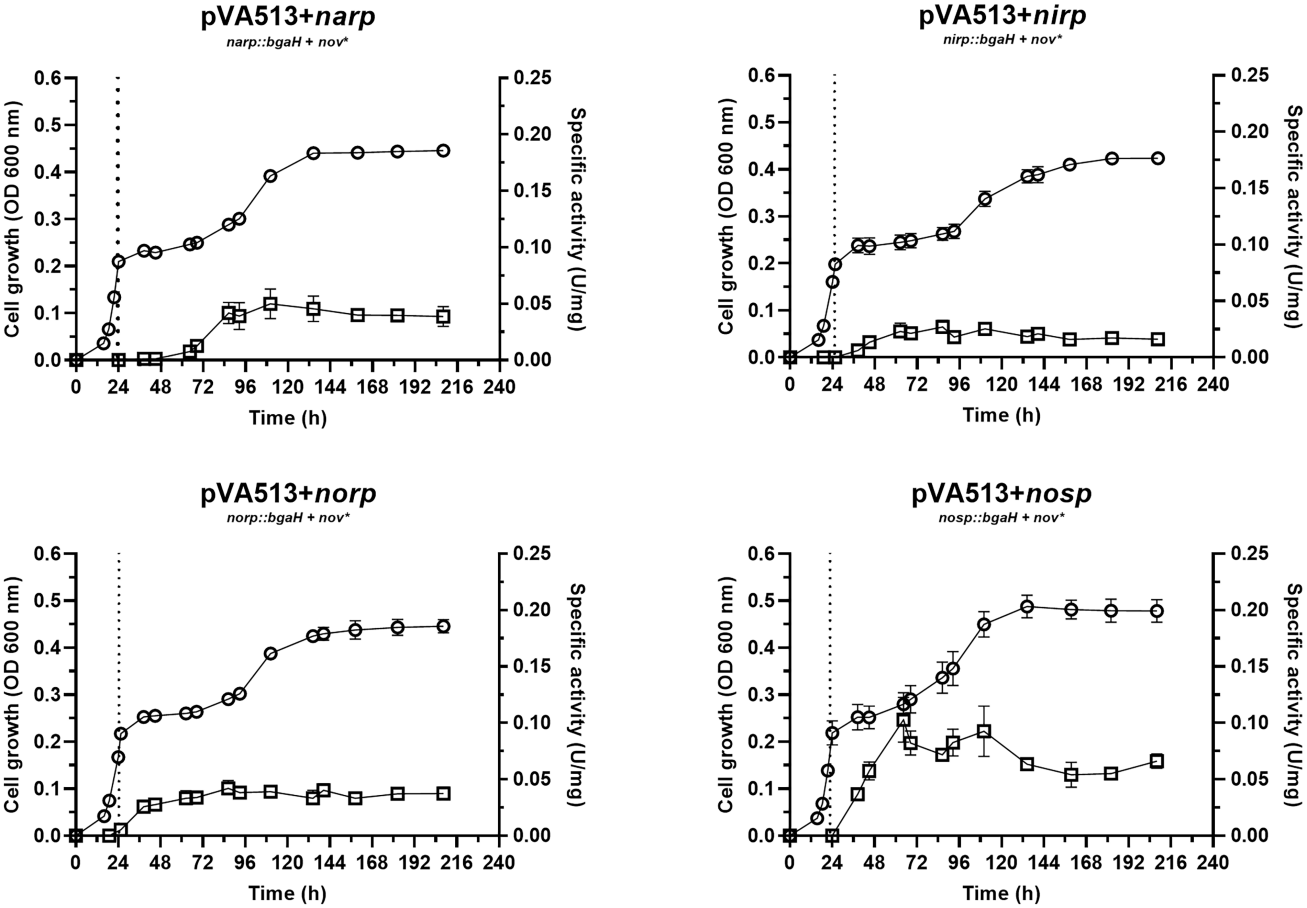
*Haloferax mediterranei* R4 anaerobic growth curves (circles) and promoter activity (squares) of the four main genes of denitrification (*narGH*, *nirK*, *nor,* and *nosZ*) in defined media supplemented with DMSO as an electron acceptor. Promoter activity was measured using the β-galactosidase specific activity. The dotted lines represent the switch from oxic conditions to anoxic. Titles of each graph represent the specific strain used for that study. pVA513 refers to the plasmid used that carries the reporter gene (*bgaH* gene). *narp* (nitrate reductase promoter), *nirp* (nitrite reductase promoter), *norp* (nitric oxide reductase promoter), and *nosp* (nitrous oxide reductase promoter) refer to the promoter cloned in the plasmid. *Nov:* novobiocin resistance.

The growth and activation of promoters under the presence of an electron acceptor other than O_2_ were first assessed in media supplemented with DMSO (Figure 2). This sulfur compound can be used by *H. mediterranei* (and many other haloarchaea) for anaerobic respiration due to the action of the DMSO reductase enzyme, which reduces DMSO to dimethyl sulfide (DMS) (Qi et al., 2015). These compounds, together with dimethylsulphoniopropionate (DMSP) are key components of the marine sulfur cycle, and some studies pointed out that saline environments are associated with high concentrations of them (Yoch, 2002; Asher et al., 2015). This association derives mainly from the plant and microbial diversity present in saline environments, which are major sources of sulfur compounds such as these (Yoch, 2002).

The growth of cells in DMSO-supplemented media showed a diauxic form with the typical three phases once cells enter anoxic conditions: lag phase in which cells are adapting to the new condition, exponential phase, and finally a stationary phase, with an estimated growth-specific velocity in the exponential phase of 0.0085 h^−1^ (Figure 2; Supplementary Figure S5; Supplementary Table S7). Regarding promoter activation (Figure 2), no significant differences for the maximum specific activities reached were found compared to cultures without electron acceptors, except for the *narp* (Supplementary Tables S1, S2). This promoter displayed a major activation when DMSO was present in the culture media compared with the cultures without electron acceptors (Table 4; Supplementary Tables S1, S2). This activation occurred approximately at the beginning of the exponential phase under anoxic conditions. DMSO respiration is also activated under anoxic conditions in presence of DMSO in *H. volcanii* cells, but, in this case, it is repressed in the presence of nitrate by a shared regulator (NarO), which acts as an activator of *nar* operon and a nitrate dependent repressor of *dms* operon (Koyanagi et al., 2021). In *H. mediterranei,* the behavior observed indicates that transcription of *nar* operon is activated in the presence of DMSO (compared with cultures without electron acceptor) and this activation has not been reported so far (Figure 2; Supplementary Tables S1, S2). Therefore, the reason explaining *narp* activation under this condition is unclear, opening new questions about the connection between DMSO respiration and denitrification.

**Table 4:** Maximum specific activity values (U/mg) reached in anaerobic cultures containing different final electron acceptors.

Electron acceptor	Condition	*narp*	*nirp*	*norp*	*nosp*
No EA*	Anoxic	0.0058 ± 0.0009	0.037 ± 0.015	0.05 ± 0.02	0.087 ± 0.013
DMSO	Anoxic	0.050 ± 0.013	0.027 ± 0.006	0.042 ± 0.007	0.10 ± 0.02
Nitrate	Anoxic	1.0 ± 0.2	3.4 ± 0.8	3.7 ± 0.2	2.2 ± 0.6
	Oxic	0.0345 ± 0.0044	0.0189 ± 0.0073	0.08525 ± 0.0042	0.0657 ± 0.011
Nitrite	Anoxic	1.3 ± 0.3	2.6 ± 0.2	2.5 ± 0.7	3.2 ± 0.6

*Narp* (nitrate reductase promoter), *nirp* (nitrite reductase promoter), *norp* (nitric oxide reductase promoter), and *nosp* (nitrous oxide reductase promoter) refer to the promoter cloned in the plasmid pVA513. ^*^No electron acceptor other than remaining O_2_.

**Figure 3:**
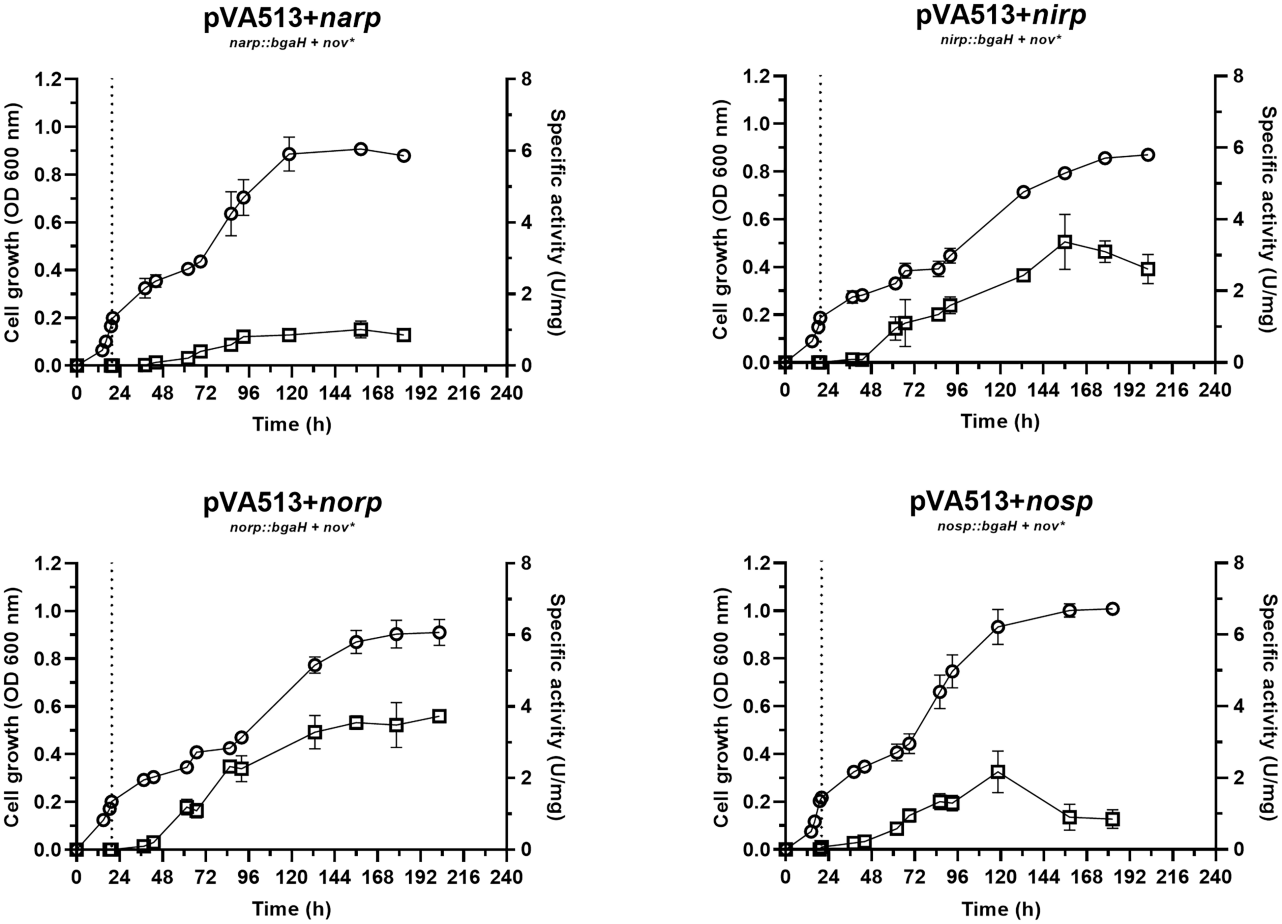
*Haloferax mediterranei* R4 anaerobic growth curves (circles) and promoter activity (squares) of the four main genes of denitrification (*narGH*, *nirK*, *nor,* and *nosZ*) in defined media supplemented with nitrate as an electron acceptor. Promoter activity was measured using the β-galactosidase specific activity. The dotted lines represent the switch from oxic conditions to anoxic. Titles of each graph represent the specific strain used for that study. pVA513 refers to the plasmid used that carries the reporter gene (*bgaH* gene). *narp* (nitrate reductase promoter), *nirp* (nitrite reductase promoter), *norp* (nitric oxide reductase promoter), and *nosp* (nitrous oxide reductase promoter) refer to the promoter cloned in the plasmid. *Nov:* novobiocin resistance.

The next final electron acceptor tested was nitrate (Figure 3). Similar denitrifying conditions (anaerobiosis and presence of nitrate) were analyzed in a previous study in complex media with yeast extract as a nutrient source, but not in defined media (Torregrosa-Crespo et al., 2020a). In the present study, temperature and media composition were adjusted to the optimal growth conditions. As a result of this optimization, the cultures reached an estimated growth-specific velocity of 0.0123 h^−1^ under denitrifying conditions (Supplementary Figure S6; Supplementary Table S7).

In terms of promoter activation, *nirp* and *norp* displayed the highest specific activity (Table 4; Supplementary Figure S4). These data are in line with transcriptomics studies in which *nirK* and *nor* genes showed higher RNA copy numbers than *nar* and *nos* (Torregrosa-Crespo et al., 2020a).

**Figure 4:**
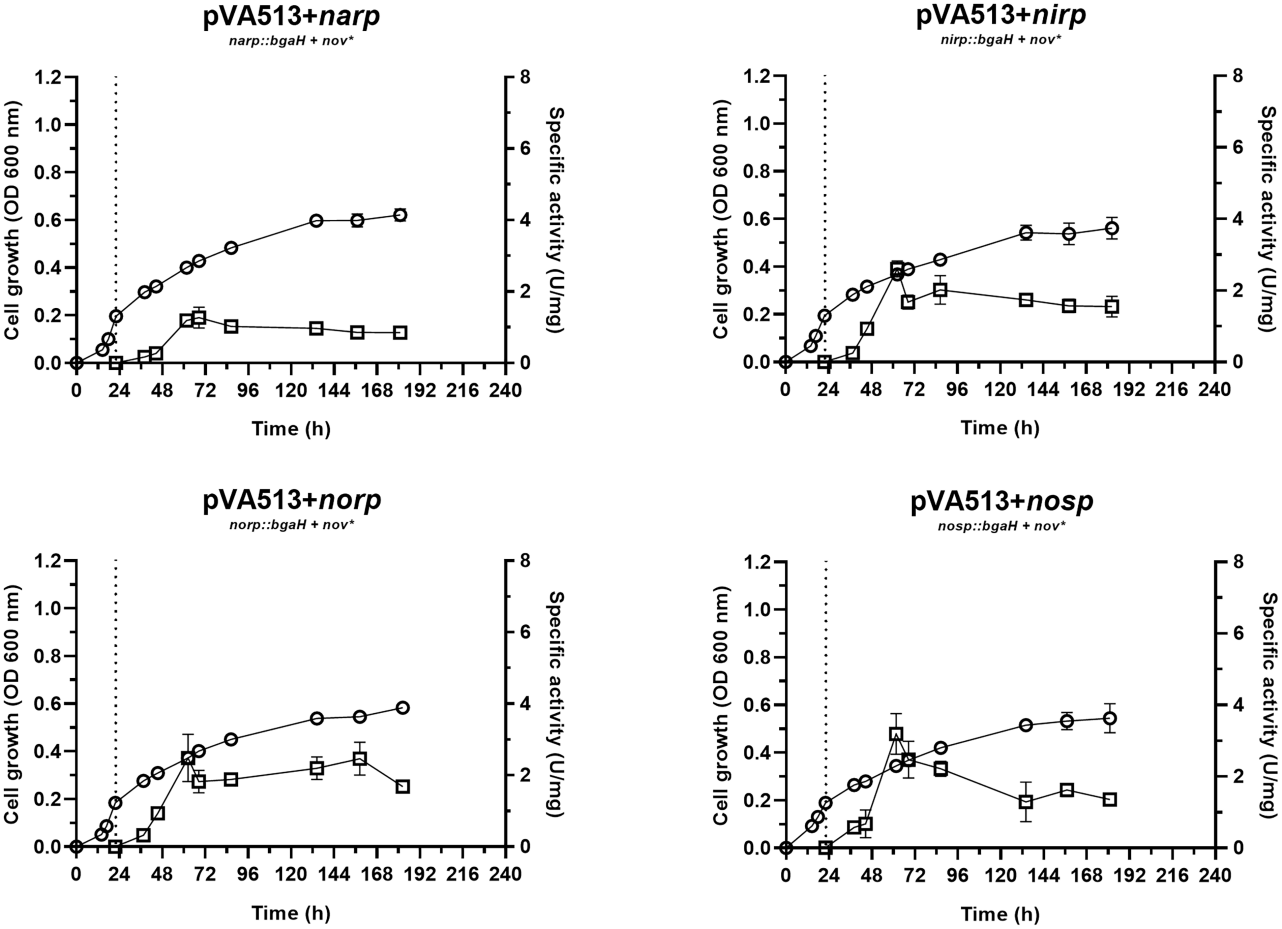
*Haloferax mediterranei* R4 anaerobic growth curves (circles) and promoter activity (squares) of the four main genes of denitrification (*narGH*, *nirK*, *nor,* and *nosZ*) in defined media supplemented with nitrite as an electron acceptor. Promoter activity was measured using the β-galactosidase specific activity. The dotted lines represent the switch from oxic conditions to anoxic. Titles of each graph represent the specific strain used for that study. pVA513 refers to the plasmid used that carries the reporter gene (*bgaH* gene). *narp* (nitrate reductase promoter), *nirp* (nitrite reductase promoter), *norp* (nitric oxide reductase promoter), and *nosp* (nitrous oxide reductase promoter) refer to the promoter cloned in the plasmid. *Nov:* novobiocin resistance.

Finally, nitrite presence as an alternative electron acceptor was tested under the same anoxic conditions (Figure 4). Denitrification using nitrite as the final electron acceptor does not have the same yield reached in the presence of nitrate, due to both, nitrite toxicity and lower proton motive force connected to ATP production. In terms of bioenergetic, nitrate reduction can move 2H^+^ to the pseudo periplasm, which are not translocated when the reduction starts from nitrite (Martinez-Espinosa et al., 2007). Growth data revealed that the estimated specific growth rate of the cells in media supplemented with nitrite was 0.0089 h^−1^ (Supplementary Figure S7; Supplementary Table S7), which is slightly lower than the one shown in the presence of nitrate. It is relevant to notice that there is almost no lag phase when cells are transferred to low oxygen conditions, may be due to faster adaptation of the cells to the anaerobic growth under this situation.

This faster adaptation could be explained by the fact that *narp* is less responsive than *nirp* promoter. Notably, *nirp* has shown significantly higher activation than *narp* promoter under the presence of nitrate and non-significant (value of p 0.0599) but higher activation under the presence of nitrite (Supplementary Figure S4; Supplementary Tables S3, S4). Furthermore, *nirp* activation is also faster (reaching higher specific activity values before *narp*), therefore *nir* gene transcription should occur earlier than *nar* (Figures 1–4; Table 4). Another interesting result obtained in these cultures is that maximum promoter activity is observed around 60 h of cell growth, sooner than in the presence of nitrate or DMSO. This distinctive feature can be explained by exploring the transcriptomic profiling of denitrification genes in *H. mediterranei* performed in previous studies together with gas kinetics (Torregrosa-Crespo et al., 2020a). In those studies, when cells are grown directly using nitrite instead of nitrate, a greater accumulation of nitric oxide is observed (compared with the use of nitrate) (Torregrosa-Crespo et al., 2020a). Nitric oxide is a very reactive molecule, and its accumulation is toxic to the cells. This toxicity increases transcript levels of denitrification genes to rapidly consume it when this molecule appears (Torregrosa-Crespo et al., 2020a). Hence, this accumulation of nitric oxide produced by the direct consumption of nitrite could lead to higher activation of the promoters, producing the observed peaks, which may be involved in cellular detoxification strategies. Thus, it is known that in bacteria, NO is not only an intermediate of denitrification, but also it is a signaling molecule that can alter gene expression and the same behavior is expected in haloarchaea according to these results (Poole, 2005).

Interestingly, another result obtained from this condition was the activation of *narp*. This activation reaches similar levels than in the presence of nitrate, despite NarGH does not have an electron acceptor to reduce under this condition. Due to the similar levels of *narp* activation under nitrite and nitrate conditions, a shared or similar activation could be taking place in both situations. Similar behavior has been described previously in the NarR regulator of *Paracoccus*, which is related to Nar activation and is induced either by nitrate or nitrite (Wood et al., 2001; Spiro, 2016). However, there are no homologs of NarR in *H. mediterranei*. Another explanation is that *narp* could respond to nitrite or nitric oxide and not to nitrate. Therefore, a basal activation of the promoter would be needed to transcript and translate NarGH, triggering the high activation of *narp* when nitrite or NO are present. Figure 1 shows low activation of *narp* when no electron acceptor is present. However, the exact reason for the behavior of this promoter is unknown, but this result together with the pattern observed under the presence of DMSO, show that *narp* has a different regulation than the other denitrification promoters.

Considering all the conditions analyzed, it can be deduced that the absence of oxygen itself is not such a strong signal for the activation of the denitrification response. Previous studies suggested that at least *narG* and *nosZ* genes were mostly responsive to hypoxia but observing Figures 1–4 it is inferred that the presence of an electron acceptor is crucial for a high activation of the denitrification promoters in *H. mediterranei,* as can be seen by comparing promoter activity in media supplemented with nitrate or nitrite with those in absence of electron acceptor (Table 4; Supplementary Figure S2; Torregrosa-Crespo et al., 2020a).

### 3.2. Promoter activation is initiated at low oxygen concentration

**Figure 5:**
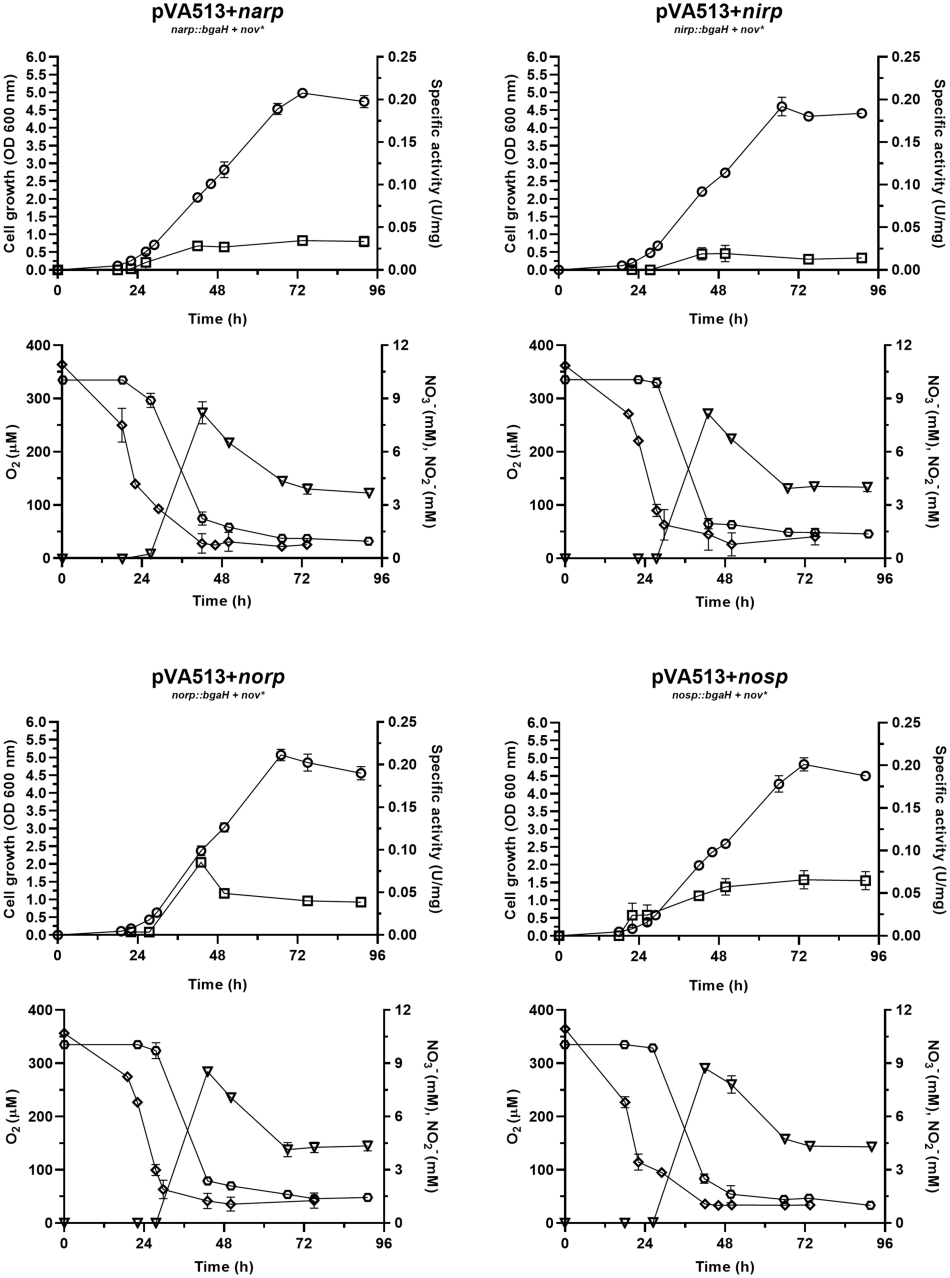
*Haloferax mediterranei* R4 aerobic growth curves (circles) and promoter activity (squares) of the four main genes of denitrification (*narGH*, *nirK*, *nor,* and *nosZ*) in defined media supplemented with nitrate as electron acceptor. Promoter activity was measured using the β-galactosidase specific activity. Below each growth graph, it is displayed the measurements of different parameters of the media: oxygen (diamond), nitrate (hexagon), and nitrite (triangles). Titles of each graph represent the specific strain used for that study. pVA513 refers to the plasmid used that carries the reporter gene (*bgaH* gene). *narp* (nitrate reductase promoter), *nirp* (nitrite reductase promoter), *norp* (nitric oxide reductase promoter), and *nosp* (nitrous oxide reductase promoter) refer to the promoter cloned in the plasmid. *Nov:* novobiocin resistance.

The presence of denitrification genes in haloarchaeal genomes represents an evolutive adaptation to their environments, where oxygen solubility is very low (Oren, 2016; Miralles-Robledillo et al., 2021). The next step in this study was to analyze whether low oxygen conditions are necessary for the activation of denitrification. Aerobic cultures supplemented with nitrate, the electron acceptor that showed a higher growth-specific velocity value, were set up. These cultures were incubated under continuous agitation, and their air chamber was continuously renewed each time samples were taken, to ensure the oxygen exchange with the media. Figure 5 displays the results obtained for each strain.

Reported data on oxygen concentration in hypersaline environments present variations, as dissolved oxygen concentration changes depending on parameters such as salinity, water movement, temperature, or depth at which the sample is taken. Therefore, oxygen concentration values in these environments vary between 2.5 and 250 μM (Rodriguez-Valera et al., 1985; Pavlova et al., 1998; Máthé et al., 2014; Oren, 2016; El-Mezayen et al., 2018). The experimental design here described showed a maximum peak of dissolved oxygen concentration around 360 μM at *t* = 0 (this high value is probably given due to the continuous agitation) and displayed a decrease reaching 25–50 μM as minimum values at ≈48 h, as the cell culture grew. This variation is caused by cell growth, as the oxygen demand increases, and because *H. mediterranei* is an exopolysaccharide-producing microorganism. This exopolysaccharide increases the viscosity of the medium contributing to the decrease in oxygen solubility, despite the continuous agitation of the medium (Antón et al., 1988; Boán et al., 1998; Chen et al., 2020). However, this oxygen concentration seems to be sufficient to maintain exponential growth throughout the experiment and to reach high OD_600nm_ values compared to cultures under fully anoxic conditions.

Data showed that during the first 27 h of growth, there is enough dissolved oxygen to sustain aerobic respiration without denitrification because there is almost no nitrate consumption and almost no promoter activation. Then, the critical oxygen concentration of ≈100 μM was reached and nitrate consumption by denitrification starts, followed by nitrite production. From this point, the promoter’s activity began to increase and nitrate, and oxygen were consumed rapidly as cell density was increasing. Nitrite production reached its maximum value at around 42.5 h, with a maximum concentration of 8.2–8.7 μM. This nitrite was subsequently consumed until cell growth reached the stationary phase. It is also noticeable that the peak of maximum activity of the *norp* appears in coordination with the maximum nitrite values, and therefore maximum production of nitric oxide. Stationary cell growth is reached at ≈74 h, and all measured values were constant due to the decrease in metabolic activity and cell death. Finally, the maximum values of activity reached in this experiment are of the same order as those obtained under anoxic conditions supplemented with DMSO and without an electron acceptor (Table 4). Therefore, under no circumstances these measurements reached the values obtained with nitrate or nitrite in anoxia (Table 4; Supplementary Figure S4).

**Figure 6:**
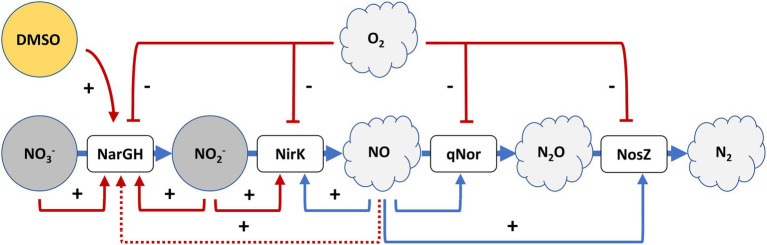
A tentative model for denitrification regulatory signals in *H. mediterranei* R4. In red are the signals that have been assessed in this paper, that complement the first proposed model. The activation of respiratory nitrate reductase transcription by NO has been proposed in this paper (dot line), but it should be analyzed with further studies. Adapted from Torregrosa-Crespo et al. (2020a).

Considering these data together with those observed under anoxic conditions, it is concluded that the activation of denitrification is mainly due to two factors that need to be combined: low oxygen availability and the presence of electron acceptors. Separately, these two elements (Figures 1, 5) cannot produce a high activation of the denitrification promoters (compared to the activation reached in anoxic media supplemented with nitrate or nitrite) but combined, the response of the main denitrification promoters is produced and the growth under denitrifying conditions is supported. The first regulatory model for denitrification was proposed by Torregrosa-Crespo and co-workers in 2020 (Torregrosa-Crespo et al., 2020a); the results here described complement it regarding the regulatory signal network that is controlling denitrification promoters (Torregrosa-Crespo et al., 2020a). An updated version of the denitrification model in haloarchaea is displayed in Figure 6. This version has added new connections between the intermediates of the pathway, such as the activation of both promoters *narp* and *nirp* in the presence of NO_2_^−^ and the demonstration of the activation of *narp* in the presence of NO_3_^−^. In addition, this model has linked the sulfur cycle with denitrification due to the activation of the *narp* by DMSO and has opened new questions about the possible role of NO in *narp* activation. To summarize, when oxygen concentration drops below ≈100 μM a small activation of denitrification response is observed if an electron acceptor is available for respiration (Figures 2–5). Under these circumstances oxygen and nitrate are concomitantly consumed, indicating that aerobic respiration should be occurring simultaneously with denitrification supporting an exponential growth, and that denitrification in *H. mediterranei* may not require a dramatic decrease in O_2_ concentration (Chen and Strous, 2013; Ji et al., 2015; Yang et al., 2020). However, it is worth mentioning that the maximum activity of the promoter is reached under completely anoxic conditions, as shown in section 3.1. Finally, it is also noticed an activity peak of the *norp* promoter coordinated with nitrite production under this culture condition, probably due to the subsequent formation of nitric oxide.

### 3.3. A candidate regulatory motif is shared by all promoter regions

**Figure 7:**
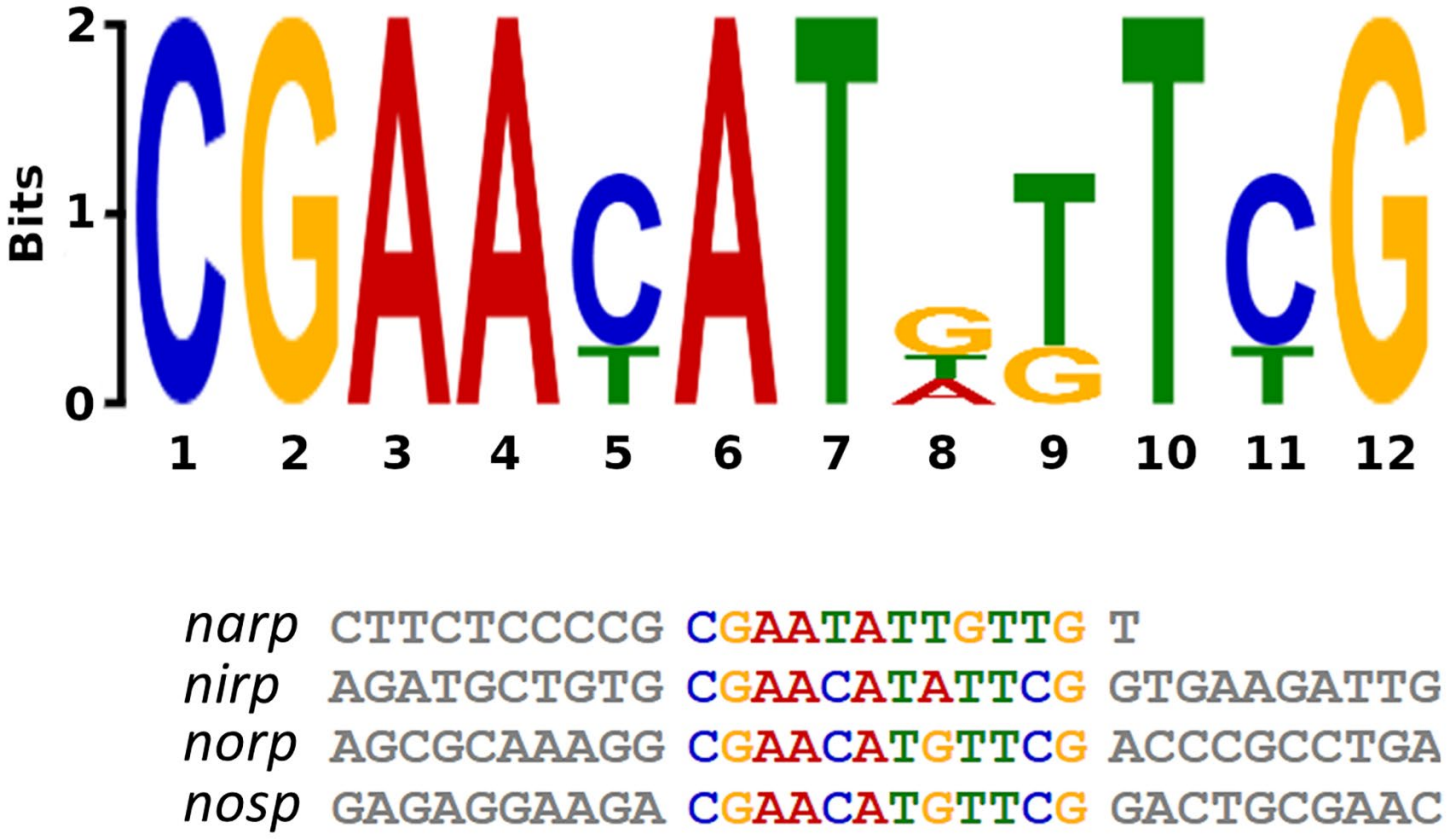
Semi-palindromic motif found in the promoter regions of the four main genes of denitrification. *Nosp*: nitrous oxide reductase promoter; *norp*: nitric oxide reductase promoter; *nirp*: nitrite reductase promoter; *narp*: nitrate reductase promoter. In *nirp*, *norp* and *nosp* promoters the motif is located at 79, 66, and 69 nucleotides upstream of the ATG initiation codon (of the gene in the case of *nirK* and *nor* and of the first gene of the operon in the case of *narGH* and *nosZ*) respectively, and in *nar* promoter is found at 319 nucleotides upstream of the initiation codon.

Genetic regulation of the denitrification pathway is not well-known in haloarchaea, whereas in bacteria there is a wide range of small-RNAs and transcriptional regulators identified alongside their mechanisms of action (Qu et al., 2016; Gaimster et al., 2018, 2019; Durand and Guillier, 2021). To better understand how promoter activation is produced in haloarchaeal denitrification, a search for common regulatory motives was carried out employing bioinformatic methods. MEME software identified a shared motif in the four promoter regions studied: CGAAYATDKTYG (Bailey et al., 2015). This motif is semi-palindromic and is especially well conserved in *nirp*, *norp,* and *nosp* promoters, whereas in *narp* has more variations (Figure 7). The identified motif has different positions in each promoter region. In *nirp*, *norp* and *nosp* promoters is located at 79, 66, and 69 nucleotides upstream of the ATG initiation codon (of the gene in the case of *nirK* and *nor* and of the first gene of the operon in the case of *narGH* and *nosZ*) respectively, but in *nar* promoter is found at 319 nucleotides upstream of the initiation codon (Supplementary Figures S2, S3). Therefore, *nirp*, *norp,* and *nosp* promoters not only share a more conserved sequence, but also a similar position of it.

**Figure 8:**
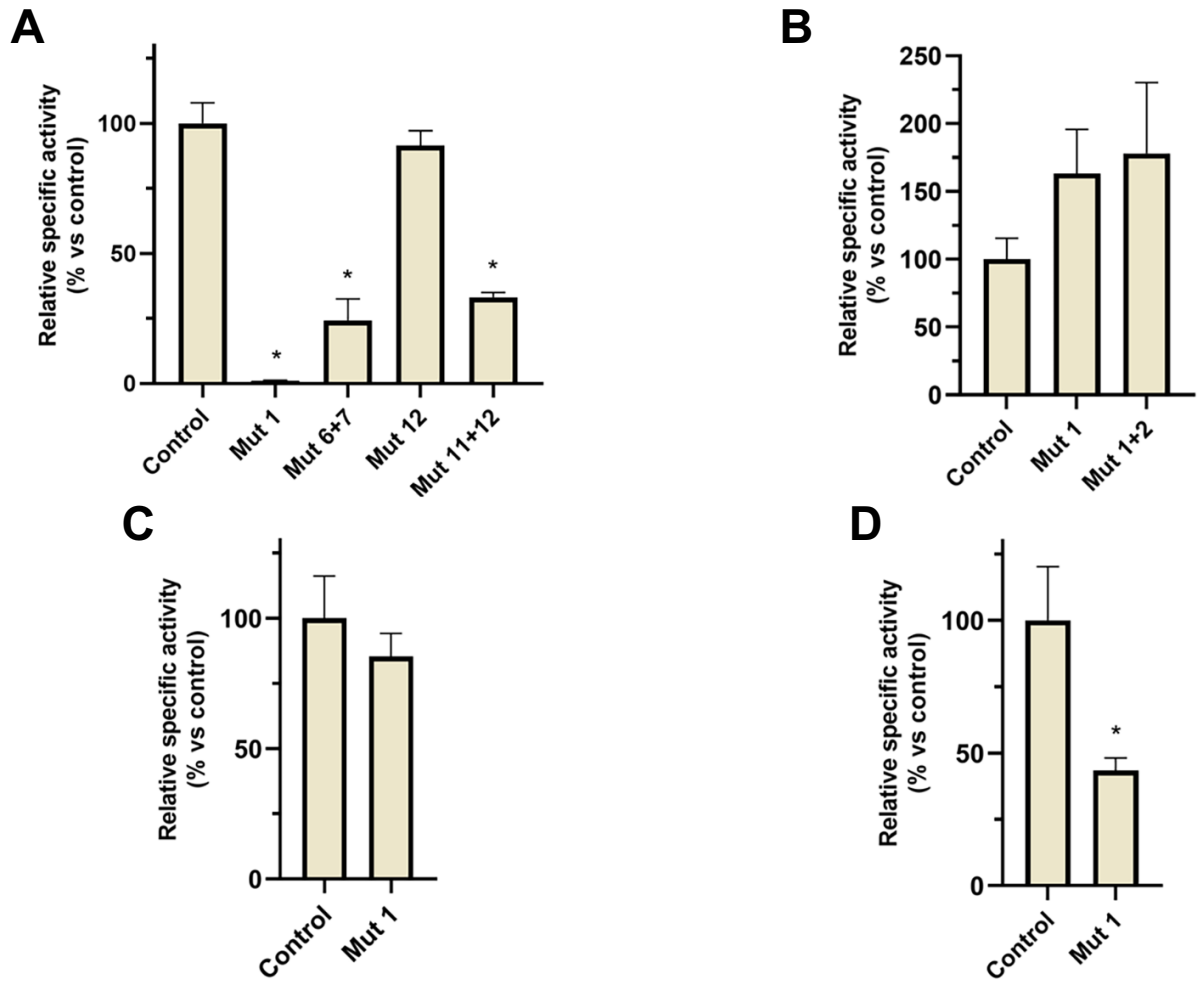
β-galactosidase relative specific activity measured in the different *H. mediterranei* R4 mutants analyzed in site-directed mutagenesis experiments (comparisons against non-mutated control strain). Cultures were grown under anaerobic conditions without an electron acceptor and measurements were taken after 24 h of anoxic growth. **(A)** *nos* promoter mutants, **(B)** *nar* promoter mutants, **(C)** *nir* promoter mutants, and **(D)** *nor* promoter mutants. ^*^Conditions with significative differences compared to control (value of *p* < 0.05).

A similar motif has been also found in the *narGH, nirK, nor, and nosZ* promoter regions of other haloarchaea from *Haloferax, Halomicrobium Halogeometricum, Halorubrum, Halorhabdus, and Haloarcula* genera (Hattori et al., 2016). However, it should be noted that in *H. mediterranei* this motif has also been found in 83 promoter regions of different genes (Supplementary Table S8). These promoter regions include the promoters of the main denitrification genes (or its operons), and genes encoding some proteins previously reported as accessory candidates involved in haloarchaeal denitrification. These accessory proteins are a multicopper oxidase domain-containing protein (NCBI gene ID: E6P09_RS00725) that could be involved in the electron transfer during nitrite/nitric oxide reduction, a hypothetical protein (NCBI gene ID: E6P09_RS00790) that is hypothesized to act as electron donor or as an oxygen scavenger, and an halocyanin (NCBI gene ID: E6P09_RS00740) which is suggested to play a role in the electron transfer during nitrite and/or nitric oxide reduction (Torregrosa-Crespo et al., 2020b). In addition, it is also noticeable that there are a high number of promoter regions of genes that encode transporters, transcriptional regulators from different families, and a truncated hemoglobin that would be proposed as accessory proteins of the denitrification metabolism. To clarify the role of this semi-palindromic sequence in the promoter regions of the main denitrification genes here studied, a site-directed mutagenesis experiment had been designed (Figures 8A–D). Firstly, the *nosp* motif, which is perfectly semi-palindromic, was mutated at different positions to find if the activity of the promoter is affected by the mutations (Table 3). The results showed that Mut 1 (mutation in the first nucleotide of the semi-palindrome) presented the most drastic change in terms of specific activity in the *nosp* promoter (Figure 8A). Moreover, the other performed mutations in the promoter also have an effect, decreasing the activity significantly, except for mutation 12 (Figure 8A). Subsequently, the rest of the promoters were also analyzed mutating at least the first nucleotide of the palindrome. *Nir* and *nor* promoters displayed a decrease in the activity, but not as drastic as *nos* promoter, and only the *norp* (besides *nosp*) decrease was statistically significant (Figures 8C,D). On the contrary, *narp* showed different behavior as the mutations displayed a nonsignificant increase in the activity of the promoter (Figure 8B). This difference could be due to the long distance of the consensus sequence to the first gene of the *nar* operon (319 base pairs upstream of the start codon) which indicates that, probably, this sequence is not controlling the expression of the *nar* operon. As well, the motif presents a high variation in the *narp* (Figure 7).

In fact, in the previously observed data (Section 3.1), *narp* showed a different activation under different conditions in comparison to the other promoters. This result may suggest that the semi-palindrome found in *narp* is regulating another genetic element, or that the semi-palindrome has not a regulatory role. Therefore, it is unlikely that the mutation of the semi-palindrome in *narp* influences promoter activation. *Narp* probably has a different genetic regulation than the other denitrification promoters. However, *nirp*, *norp,* and *nosp* promoters show more similarities regarding their activation pattern and in the location and functionality of this semi-palindromic motif (despite the *nirp* mutation was not statistically significant). Thus, these three promoters might share a common regulator.

## 4. Conclusion

Denitrification in haloarchaea is a poorly understood pathway compared to bacterial denitrification. Nevertheless, the last studies have pointed out that denitrification in hypersaline environments could be a source of nitrogenous gasses to the atmosphere (mainly if partial denitrification predominates vs. complete denitrification), and greater efforts should be made to deepen the knowledge of these extreme environments (Miralles-Robledillo et al., 2021). In this context, this work represents an advance in the understanding of the external signals that can modulate the activation of this alternative respiration. The presence of electron acceptors and low oxygen conditions have been found to be essential signals for the activation of denitrification. Moreover, these conditions must occur together, since individually they are not capable of producing a high activation of any of the promoters of the main enzymes involved in the process. Another important feature observed in the cultures with oxygen is that denitrification begins with oxygen present in the media (≈100 μM). This data supports the patterns proposed in previous work: induction of denitrification in *H. mediterranei* does not require full anaerobiosis and it is rather a parallel process that can occur at the same time as aerobic respiration if there is low oxygen availability in the environment (Chen and Strous, 2013; Torregrosa-Crespo et al., 2020a). Finally, it has been concluded that at least *norp, nosp* and probably *nirp* could have common transcriptional regulators because of the presence of a well-conserved motif in their sequence and similar promoter activation patterns. The *narp* promoter also carries a similar motif, but it is not as conserved or in the same position as in the other promoters. Therefore, these data, together with the promoter activity under different conditions, suggest that a distinct regulatory mechanism should control the activity of the *narp* promoter. In addition, this motif has also been found in the promoter region of other proteins previously suggested as accessory proteins of the denitrification pathway in haloarchaea, indicating that this motif could be the operator of a denitrification regulon.

In summary, the genetic regulation driving denitrification in haloarchaea appears to be like the bacterial counterpart. Negative regulation of denitrification by O_2_ and shared regulators for different genes acting as sensors for *N*-species are common in denitrifying bacteria (Spiro, 2016). However, in haloarchaea, these regulators remain unknown. The next step in this research is to look for key transcriptional regulators that are under this regulation. Therefore, further research using different approaches is needed to find new potential regulators.

## Data availability statement

The original contributions presented in the study are included in the article/Supplementary material, further inquiries can be directed to the corresponding author.

## Author contributions

CP and RM-E conceived the project and managed the funding. JM-R, CP, and RM-E conceived and designed the study, analyzed the results, and contributed equally to the writing and review of the original manuscript. JM-R conducted the experiments and data analysis. All authors contributed to the article and approved the submitted version.

## Funding

This work was funded by MINECO, Spain (2019/00673/001), Generalitat Valenciana, Spain (PROMETEO/2021/055), and VIGROB-309 (University of Alicante).

## Conflict of interest

The authors declare that the research was conducted in the absence of any commercial or financial relationships that could be construed as a potential conflict of interest.

## Publisher’s note

All claims expressed in this article are solely those of the authors and do not necessarily represent those of their affiliated organizations, or those of the publisher, the editors and the reviewers. Any product that may be evaluated in this article, or claim that may be made by its manufacturer, is not guaranteed or endorsed by the publisher.

## Footnotes

^1^ http://meme-suite.org/

## Abbreviations

*narGH*: nitrate reductase
*nirK*: nitrite reductase
*nor*: nitric oxide reductase
*nosZ*: nitrous oxide reductase
*narp*: promoter region of nitrate reductase operon
*nirp*: promoter region of the nitrite reductase gene
*norp*: promoter region of the nitric oxide reductase gene
*nosp*: promoter region of the nitrous oxide reductase operon
OD_600 nm_: Optical density value at 600 nanometers
NEDA: N-(1-naphthyl)-ethylenediamine-dihydrochloride
Abs: Absorbance
DMSO: Dimethyl sulfoxide
MOPS: (3-(N-morpholino) propanesulfonic acid
ONPG: ortho-nitro-phenol β-D-galactoside
ONP: o-nitrophenol
