# Supplementary material for: Analysis of the external signals driving the transcriptional regulation of the main genes involved in denitrification in Haloferax mediterranei
Source: Front Microbiol. 2023 Mar 16;14:1109550. doi: 10.3389/fmicb.2023.1109550 (PMC10062603; doi:10.3389/fmicb.2023.1109550)
Supplement: Supplementary file 1 [file Data_Sheet_1.docx]

Supplementary Material

**A**


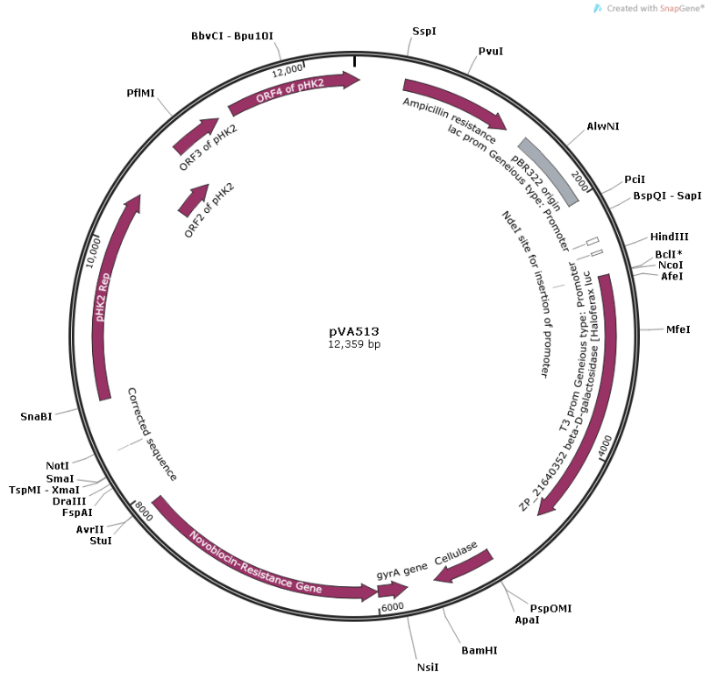


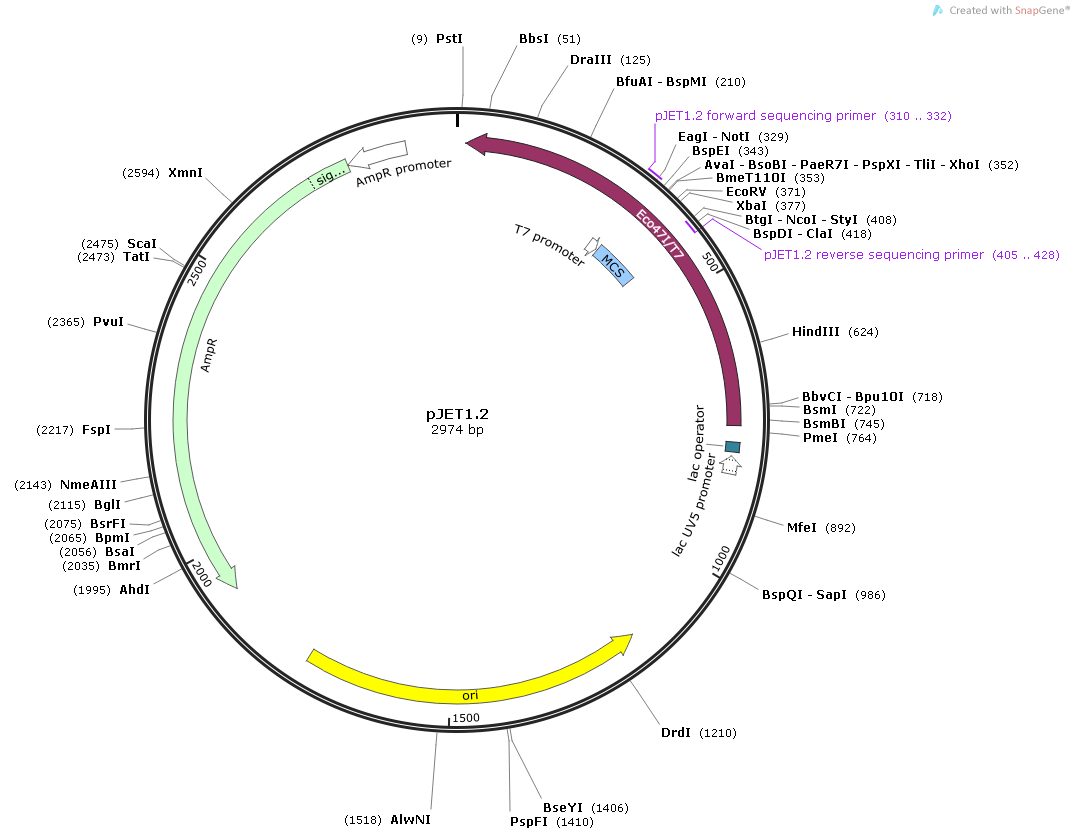
**B**

**Supplementary Figure 1.** Vector Map of the plasmids used in this study. A) pJet1.2 B) pVA513. Pictures were created with SnapGene® software (from Insightful Science; available at snapgene.com).

**
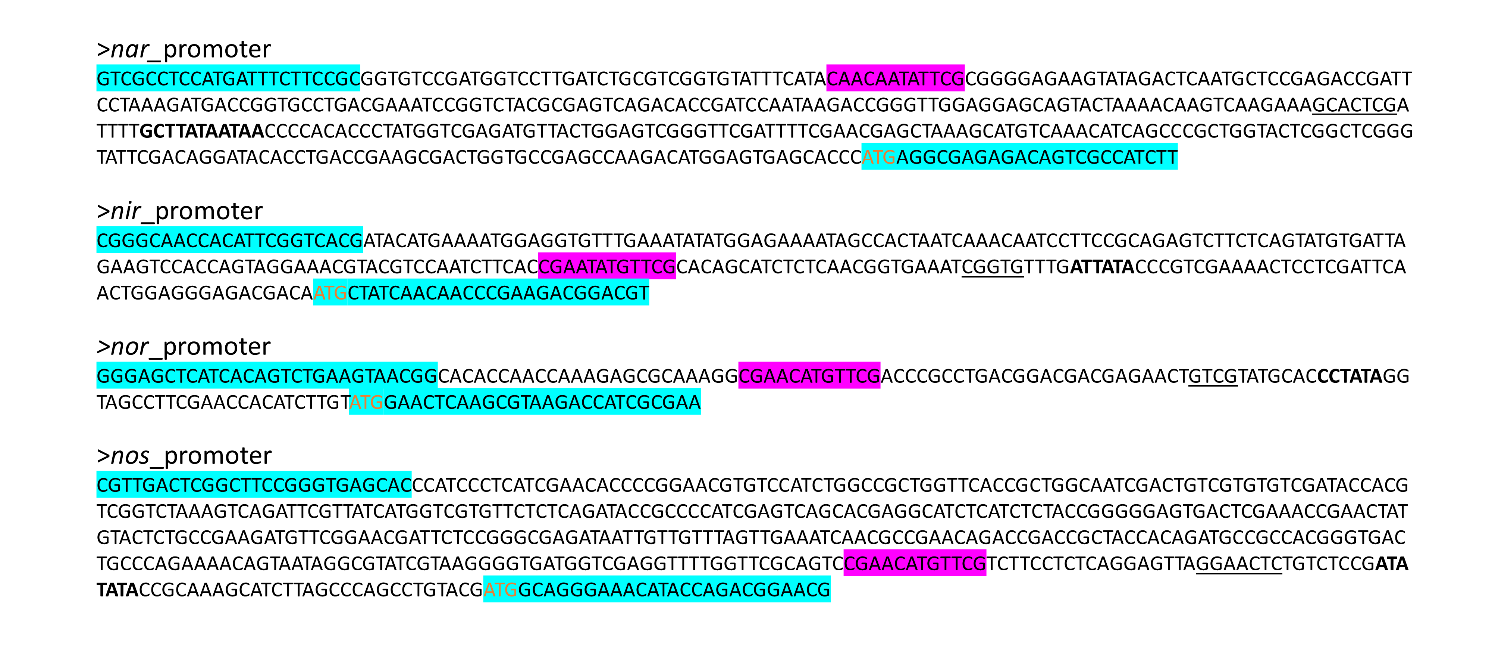
**

**Supplementary Figure 2.** Sequence of the promoter regions analyzed in this study. In purple, the semi-palindromic sequence found in the promoters, in orange, the start ATG of the corresponding gene, and in blue, the sequences where the primers hybridize. Bold nucleotides indicate the area where the possible TATA box was identified, and underlined nucleotides indicate the region where the possible BRE box was identified.

**
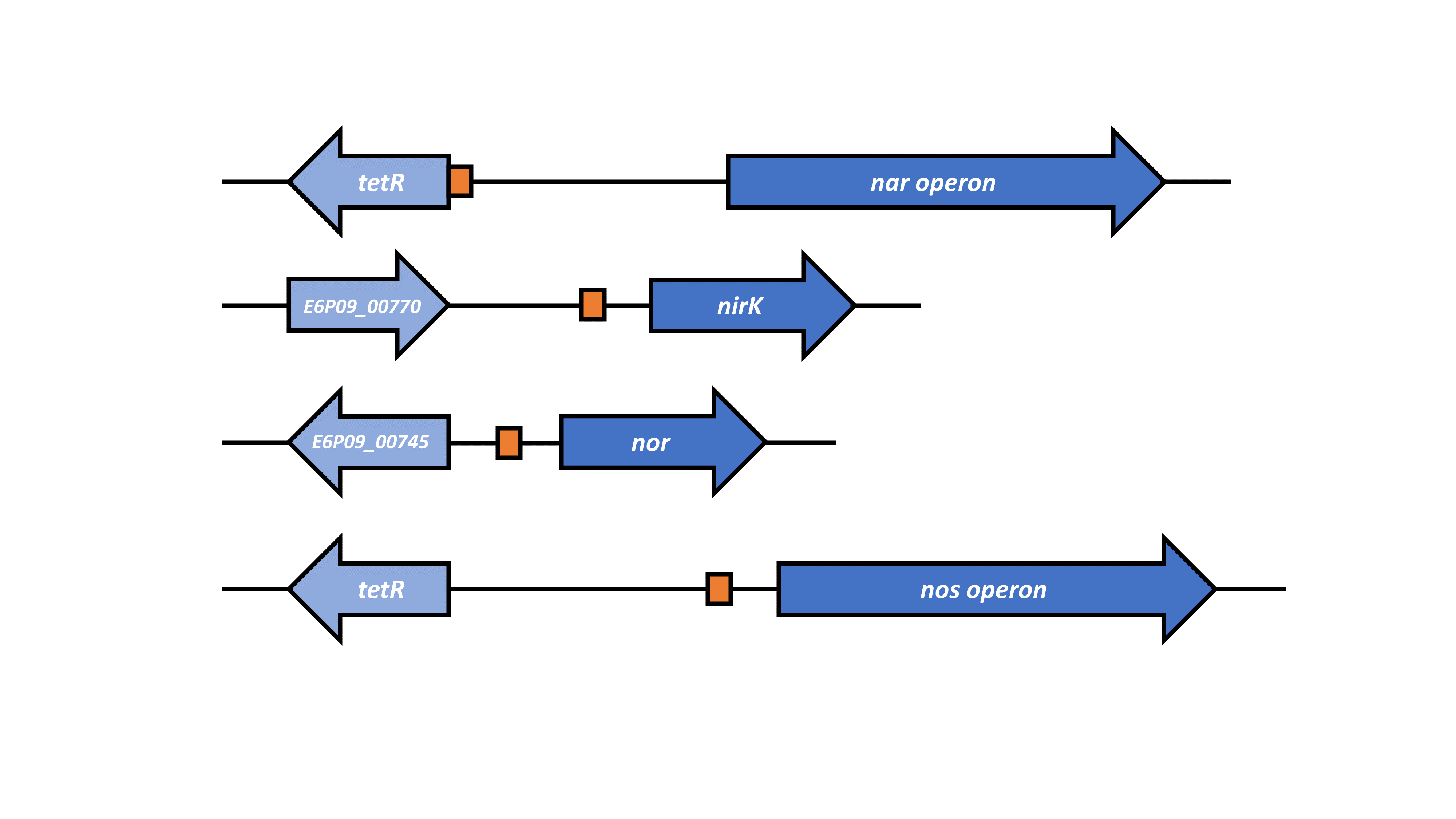
**

**Supplementary Figure 3.** Promoter regions studied in this research (*narp:* 420 bp; *nirp*: 267 bp; *norp*: 159 bp; *nosp*: 501 bp). Red boxes represent the location of the semi-palindromic motif (distance from the motif to the start codon of the denitrification gene is: 319 bp for *narp,* 79 bp for *nirp*, 66 bp for *norp,* and 69 bp for *nosp*). Figure distances are approximations.

**
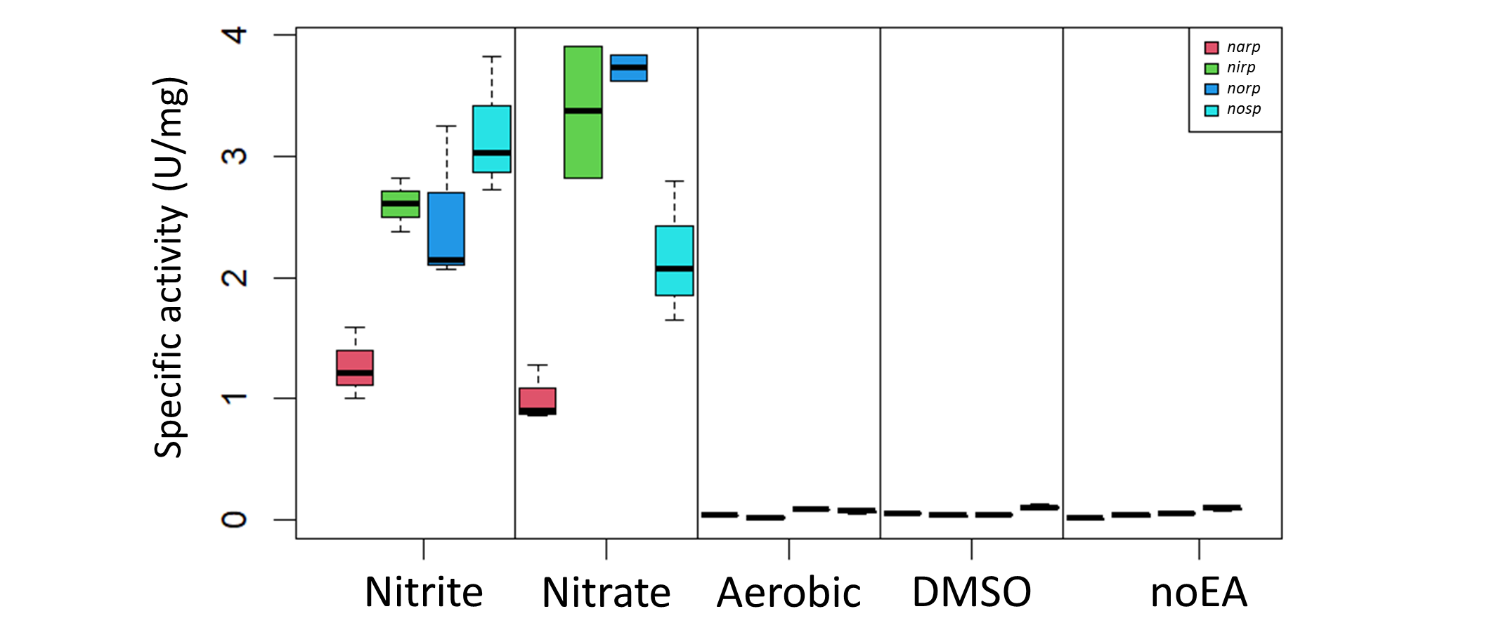
**

**Supplementary Figure 4**. Box-and-whisker plot of the specific β-galactosidase activity measurements (U/mg) of the four tested promoters (*narp*: nitrate reductase promoter, *nirp*: nitrite reductase promoter, *norp*: nitric oxide reductase promoter and *nosp*: nitrous oxide reductase promoter) obtained under different conditions. noEA: No electron acceptor other than remaining O_2_.

**Supplementary Table 1**. Summary of the two-way ANOVA of the identification of significant differences in the maximum promoter activities (specific activity) of the different promoters among the anaerobic cultures supplemented with DMSO (DMSO), the anaerobic cultures with no electron acceptor other than remaining O2 (noEA) and the aerobic cultures supplemented with nitrate (Aerobic).

| **Two-way ANOVA** | Ordinary |  |  |  |  |
| --- | --- | --- | --- | --- | --- |
| **Alpha** | 0,05 |  |  |  |  |
| **Source of Variation** | **% of total variation** | **P value** | **P value summary** | **Significant?** |  |
| Interaction | 23,37 | <0,0001 | **** | Yes |  |
| Promoter | 63,31 | <0,0001 | **** | Yes |  |
| Condition | 1,719 | 0,1810 | ns | No |  |
| **ANOVA table** | **SS (Type III)** | **DF** | **MS** | **F (DFn, DFd)** | **P value** |
| Interaction | 0,007095 | 6 | 0,001183 | F (6, 22) = 8,381 | P<0,0001 |
| Promoter | 0,01922 | 3 | 0,006407 | F (3, 22) = 45,41 | P<0,0001 |
| Condition | 0,0005218 | 2 | 0,0002609 | F (2, 22) = 1,849 | P=0,1810 |
| Residual | 0,003104 | 22 | 0,0001411 |  |  |

**Supplementary Table 2.** Results of Tukey's multiple comparisons test to identify maximum activity differences among the anaerobic cultures supplemented with DMSO (DMSO), the anaerobic cultures with no electron acceptor other than remaining O2 (noEA) and, the aerobic cultures supplemented with nitrate (Aerobic). Alpha = 0.05. CI: confidence interval.

| **Tukey's multiple comparisons tests** | **Mean Diff,** | **95,00% CI of diff,** | **Significant?** | **Summary** | **Adjusted P Value** |
| --- | --- | --- | --- | --- | --- |
| narp:No EA vs. narp:DMSO | -0,04400 | -0,07928 to -0,008721 | Yes | ** | 0,0069 |
| narp:No EA vs. narp:Aerobic | -0,02857 | -0,06385 to 0,006712 | No | ns | 0,1914 |
| narp:No EA vs. nirp:No EA | -0,03151 | -0,07095 to 0,007934 | No | ns | 0,2051 |
| narp:No EA vs. nirp:DMSO | -0,02101 | -0,05629 to 0,01427 | No | ns | 0,5868 |
| narp:No EA vs. nirp:Aerobic | -0,01337 | -0,04865 to 0,02191 | No | ns | 0,9561 |
| narp:No EA vs. norp:No EA | -0,04663 | -0,08191 to -0,01135 | Yes | ** | 0,0037 |
| narp:No EA vs. norp:DMSO | -0,03606 | -0,07134 to -0,0007775 | Yes | * | 0,0422 |
| narp:No EA vs. norp:Aerobic | -0,07932 | -0,1188 to -0,03987 | Yes | **** | <0,0001 |
| narp:No EA vs. nosp:No EA | -0,08137 | -0,1166 to -0,04609 | Yes | **** | <0,0001 |
| narp:No EA vs. nosp:DMSO | -0,09687 | -0,1321 to -0,06159 | Yes | **** | <0,0001 |
| narp:No EA vs. nosp:Aerobic | -0,05977 | -0,09505 to -0,02449 | Yes | *** | 0,0002 |
| narp:DMSO vs. narp:Aerobic | 0,01543 | -0,01985 to 0,05071 | No | ns | 0,8948 |
| narp:DMSO vs. nirp:No EA | 0,01249 | -0,02695 to 0,05194 | No | ns | 0,9878 |
| narp:DMSO vs. nirp:DMSO | 0,02300 | -0,01229 to 0,05828 | No | ns | 0,4616 |
| narp:DMSO vs. nirp:Aerobic | 0,03063 | -0,004647 to 0,06591 | No | ns | 0,1300 |
| narp:DMSO vs. norp:No EA | -0,002626 | -0,03791 to 0,03265 | No | ns | >0,9999 |
| narp:DMSO vs. norp:DMSO | 0,007944 | -0,02734 to 0,04322 | No | ns | 0,9993 |
| narp:DMSO vs. norp:Aerobic | -0,03532 | -0,07476 to 0,004128 | No | ns | 0,1077 |
| narp:DMSO vs. nosp:No EA | -0,03737 | -0,07265 to -0,002087 | Yes | * | 0,0317 |
| narp:DMSO vs. nosp:DMSO | -0,05287 | -0,08815 to -0,01759 | Yes | *** | 0,0009 |
| narp:DMSO vs. nosp:Aerobic | -0,01577 | -0,05105 to 0,01951 | No | ns | 0,8817 |
| narp:Aerobic vs. nirp:No EA | -0,002942 | -0,04239 to 0,03650 | No | ns | >0,9999 |
| narp:Aerobic vs. nirp:DMSO | 0,007562 | -0,02772 to 0,04284 | No | ns | 0,9996 |
| narp:Aerobic vs. nirp:Aerobic | 0,01520 | -0,02008 to 0,05048 | No | ns | 0,9035 |
| narp:Aerobic vs. norp:No EA | -0,01806 | -0,05334 to 0,01722 | No | ns | 0,7689 |
| narp:Aerobic vs. norp:DMSO | -0,007489 | -0,04277 to 0,02779 | No | ns | 0,9996 |
| narp:Aerobic vs. norp:Aerobic | -0,05075 | -0,09019 to -0,01131 | Yes | ** | 0,0050 |
| narp:Aerobic vs. nosp:No EA | -0,05280 | -0,08808 to -0,01752 | Yes | *** | 0,0009 |
| narp:Aerobic vs. nosp:DMSO | -0,06830 | -0,1036 to -0,03302 | Yes | **** | <0,0001 |
| narp:Aerobic vs. nosp:Aerobic | -0,03120 | -0,06648 to 0,004080 | No | ns | 0,1163 |
| nirp:No EA vs. nirp:DMSO | 0,01050 | -0,02894 to 0,04995 | No | ns | 0,9970 |
| nirp:No EA vs. nirp:Aerobic | 0,01814 | -0,02130 to 0,05759 | No | ns | 0,8621 |
| nirp:No EA vs. norp:No EA | -0,01512 | -0,05456 to 0,02433 | No | ns | 0,9528 |
| nirp:No EA vs. norp:DMSO | -0,004548 | -0,04399 to 0,03490 | No | ns | >0,9999 |
| nirp:No EA vs. norp:Aerobic | -0,04781 | -0,09102 to -0,004599 | Yes | * | 0,0218 |
| nirp:No EA vs. nosp:No EA | -0,04986 | -0,08930 to -0,01041 | Yes | ** | 0,0060 |
| nirp:No EA vs. nosp:DMSO | -0,06536 | -0,1048 to -0,02591 | Yes | *** | 0,0002 |
| nirp:No EA vs. nosp:Aerobic | -0,02826 | -0,06770 to 0,01119 | No | ns | 0,3330 |
| nirp:DMSO vs. nirp:Aerobic | 0,007638 | -0,02764 to 0,04292 | No | ns | 0,9995 |
| nirp:DMSO vs. norp:No EA | -0,02562 | -0,06090 to 0,009660 | No | ns | 0,3155 |
| nirp:DMSO vs. norp:DMSO | -0,01505 | -0,05033 to 0,02023 | No | ns | 0,9088 |
| nirp:DMSO vs. norp:Aerobic | -0,05831 | -0,09776 to -0,01887 | Yes | ** | 0,0010 |
| nirp:DMSO vs. nosp:No EA | -0,06036 | -0,09564 to -0,02508 | Yes | *** | 0,0001 |
| nirp:DMSO vs. nosp:DMSO | -0,07586 | -0,1111 to -0,04058 | Yes | **** | <0,0001 |
| nirp:DMSO vs. nosp:Aerobic | -0,03876 | -0,07404 to -0,003481 | Yes | * | 0,0232 |
| nirp:Aerobic vs. norp:No EA | -0,03326 | -0,06854 to 0,002022 | No | ns | 0,0767 |
| nirp:Aerobic vs. norp:DMSO | -0,02269 | -0,05797 to 0,01259 | No | ns | 0,4803 |
| nirp:Aerobic vs. norp:Aerobic | -0,06595 | -0,1054 to -0,02651 | Yes | *** | 0,0002 |
| nirp:Aerobic vs. nosp:No EA | -0,06800 | -0,1033 to -0,03272 | Yes | **** | <0,0001 |
| nirp:Aerobic vs. nosp:DMSO | -0,08350 | -0,1188 to -0,04822 | Yes | **** | <0,0001 |
| nirp:Aerobic vs. nosp:Aerobic | -0,04640 | -0,08168 to -0,01112 | Yes | ** | 0,0039 |
| norp:No EA vs. norp:DMSO | 0,01057 | -0,02471 to 0,04585 | No | ns | 0,9920 |
| norp:No EA vs. norp:Aerobic | -0,03269 | -0,07214 to 0,006754 | No | ns | 0,1693 |
| norp:No EA vs. nosp:No EA | -0,03474 | -0,07002 to 0,0005383 | No | ns | 0,0561 |
| norp:No EA vs. nosp:DMSO | -0,05024 | -0,08552 to -0,01496 | Yes | ** | 0,0016 |
| norp:No EA vs. nosp:Aerobic | -0,01314 | -0,04842 to 0,02214 | No | ns | 0,9608 |
| norp:DMSO vs. norp:Aerobic | -0,04326 | -0,08271 to -0,003816 | Yes | * | 0,0235 |
| norp:DMSO vs. nosp:No EA | -0,04531 | -0,08059 to -0,01003 | Yes | ** | 0,0051 |
| norp:DMSO vs. nosp:DMSO | -0,06081 | -0,09609 to -0,02553 | Yes | *** | 0,0001 |
| norp:DMSO vs. nosp:Aerobic | -0,02371 | -0,05899 to 0,01157 | No | ns | 0,4190 |
| norp:Aerobic vs. nosp:No EA | -0,002051 | -0,04150 to 0,03739 | No | ns | >0,9999 |
| norp:Aerobic vs. nosp:DMSO | -0,01755 | -0,05699 to 0,02189 | No | ns | 0,8845 |
| norp:Aerobic vs. nosp:Aerobic | 0,01955 | -0,01989 to 0,05899 | No | ns | 0,8004 |
| nosp:No EA vs. nosp:DMSO | -0,01550 | -0,05078 to 0,01978 | No | ns | 0,8923 |
| nosp:No EA vs. nosp:Aerobic | 0,02160 | -0,01368 to 0,05688 | No | ns | 0,5488 |
| nosp:DMSO vs. nosp:Aerobic | 0,03710 | 0,001820 to 0,07238 | Yes | * | 0,0336 |

**Supplementary Table 3.** Summary of the two-way ANOVA for the identification of significant differences in the maximum promoter activities (specific activity) of the different promoters between the anaerobic cultures supplemented with nitrate or nitrite.

| **Two-way ANOVA** | Ordinary |  |  |  |  |
| --- | --- | --- | --- | --- | --- |
| **Alpha** | 0,05 |  |  |  |  |
| **Source of Variation** | **% of total variation** | **P value** | **P value summary** | **Significant?** |  |
| Interaction | 20,41 | 0,0072 | ** | Yes |  |
| Promoter | 68,88 | <0,0001 | **** | Yes |  |
| Condition | 0,8767 | 0,3909 | ns | No |  |
| **ANOVA table** | **SS (Type III)** | **DF** | **MS** | **F (DFn, DFd)** | **P value** |
| Interaction | 4,166 | 3 | 1,389 | F (3, 14) = 6,083 | P=0,0072 |
| Promoter | 14,06 | 3 | 4,686 | F (3, 14) = 20,53 | P<0,0001 |
| Condition | 0,1789 | 1 | 0,1789 | F (1, 14) = 0,7839 | P=0,3909 |
| Residual | 3,196 | 14 | 0,2283 |  |  |

**Supplementary Table 4.** Results of Tukey's multiple comparisons test to identify maximum activity differences between cultures supplemented with nitrate or nitrite. Alpha = 0.05. CI: confidence interval.

| **Tukey's multiple comparisons tests** | **Mean Diff,** | **95,00% CI of diff,** | **Significant?** | **Summary** | **Adjusted P Value** |
| --- | --- | --- | --- | --- | --- |
| narp:Nitrate vs. narp:Nitrite | -0,2565 | -1,633 to 1,120 | No | ns | 0,9970 |
| narp:Nitrate vs. nirp:Nitrate | -2,358 | -3,897 to -0,8188 | Yes | ** | 0,0018 |
| narp:Nitrate vs. nirp:Nitrite | -1,593 | -2,969 to -0,2163 | Yes | * | 0,0185 |
| narp:Nitrate vs. norp:Nitrate | -2,719 | -4,258 to -1,180 | Yes | *** | 0,0004 |
| narp:Nitrate vs. norp:Nitrite | -1,476 | -2,852 to -0,09944 | Yes | * | 0,0318 |
| narp:Nitrate vs. nosp:Nitrate | -1,159 | -2,536 to 0,2175 | No | ns | 0,1300 |
| narp:Nitrate vs. nosp:Nitrite | -2,178 | -3,554 to -0,8012 | Yes | ** | 0,0013 |
| narp:Nitrite vs. nirp:Nitrate | -2,101 | -3,640 to -0,5623 | Yes | ** | 0,0049 |
| narp:Nitrite vs. nirp:Nitrite | -1,336 | -2,713 to 0,04023 | No | ns | 0,0599 |
| narp:Nitrite vs. norp:Nitrate | -2,462 | -4,001 to -0,9233 | Yes | ** | 0,0012 |
| narp:Nitrite vs. norp:Nitrite | -1,219 | -2,596 to 0,1571 | No | ns | 0,1004 |
| narp:Nitrite vs. nosp:Nitrate | -0,9025 | -2,279 to 0,4740 | No | ns | 0,3503 |
| narp:Nitrite vs. nosp:Nitrite | -1,921 | -3,298 to -0,5447 | Yes | ** | 0,0041 |
| nirp:Nitrate vs. nirp:Nitrite | 0,7650 | -0,7740 to 2,304 | No | ns | 0,6566 |
| nirp:Nitrate vs. norp:Nitrate | -0,3611 | -2,047 to 1,325 | No | ns | 0,9930 |
| nirp:Nitrate vs. norp:Nitrite | 0,8819 | -0,6571 to 2,421 | No | ns | 0,5017 |
| nirp:Nitrate vs. nosp:Nitrate | 1,199 | -0,3402 to 2,738 | No | ns | 0,1858 |
| nirp:Nitrate vs. nosp:Nitrite | 0,1801 | -1,359 to 1,719 | No | ns | 0,9998 |
| nirp:Nitrite vs. norp:Nitrate | -1,126 | -2,665 to 0,4130 | No | ns | 0,2397 |
| nirp:Nitrite vs. norp:Nitrite | 0,1169 | -1,260 to 1,493 | No | ns | >0,9999 |
| nirp:Nitrite vs. nosp:Nitrate | 0,4338 | -0,9427 to 1,810 | No | ns | 0,9435 |
| nirp:Nitrite vs. nosp:Nitrite | -0,5849 | -1,961 to 0,7916 | No | ns | 0,7961 |
| norp:Nitrate vs. norp:Nitrite | 1,243 | -0,2961 to 2,782 | No | ns | 0,1583 |
| norp:Nitrate vs. nosp:Nitrate | 1,560 | 0,02084 to 3,099 | Yes | * | 0,0459 |
| norp:Nitrate vs. nosp:Nitrite | 0,5412 | -0,9979 to 2,080 | No | ns | 0,9059 |
| norp:Nitrite vs. nosp:Nitrate | 0,3169 | -1,060 to 1,693 | No | ns | 0,9894 |
| norp:Nitrite vs. nosp:Nitrite | -0,7018 | -2,078 to 0,6748 | No | ns | 0,6305 |
| nosp:Nitrate vs. nosp:Nitrite | -1,019 | -2,395 to 0,3578 | No | ns | 0,2293 |

**Supplementary Table 5.** Summary of the one-way ANOVA for the identification of significant differences in the promoter activities (specific activity) of the different promoters at 45 hours in anaerobic cultures without an electron acceptor other than O_2._

| **ANOVA table** | **SS** | **DF** | **MS** | **F (DFn, DFd)** | **P value** |
| --- | --- | --- | --- | --- | --- |
| Treatment (between columns) | 0,004227 | 3 | 0,001409 | F (3, 8) = 21,89 | P=0,0003 |
| Residual (within columns) | 0,0005150 | 8 | 6,437e-005 |  |  |
| Total | 0,004742 | 11 |  |  |  |

**Supplementary Table 6.** Results of Dunnett’s multiple comparisons test to identify significant differences in the promoter activities (specific activity) of the different promoters at 45 hours in anaerobic cultures without electron acceptor other than O_2._ Alpha = 0.05.

| **Dunnett's multiple comparisons tests** | **Mean Diff,** | **95,00% CI of diff,** | **Significant?** | **Summary** | **Adjusted P Value** | **A-?** |  |
| --- | --- | --- | --- | --- | --- | --- | --- |
| narp vs. nirp | -0,01930 | -0,03817 to -0,0004379 | Yes | * | 0,0453 | B | nirp |
| narp vs. norp | -0,02956 | -0,04843 to -0,01070 | Yes | ** | 0,0051 | C | norp |
| narp vs. nosp | -0,05204 | -0,07090 to -0,03317 | Yes | *** | 0,0001 | D | nosp |

**Supplementary Table 7.** Summary of growth data obtained from exponential phase semi-log plots of anaerobic cultures supplemented with different electron acceptors. CI: confidence interval.

|  | **Strain** | | | |  |  |
| --- | --- | --- | --- | --- | --- | --- |
|  | **pVA513+*narp*** | **pVA513+*nirp*** | **pVA513+*norp*** | **pVA513+*nosp*** |  |  |
|  | **Slope±Error** | **Slope±Error** | **Slope±Error** | **Slope±Error** | **Mean** | **95% CI** |
| **DMSO** | 0.0092±0.0017 | 0.0085±0.0012 | 0.0083±0.0017 | 0.0080±0.0017 | 0.0085 | 0.0077 to 0.0093 |
| **Nitrate** | 0.014±0.003 | 0.0101±0.0013 | 0.0105±0.0011 | 0.015±0.003 | 0.0123 | 0.0086 to 0.0159 |
| **Nitrite** | 0.0097±0.0013 | 0.0073±0.0007 | 0.0090±0.0009 | 0.0097±0.0010 | 0.0089 | 0.0071 to 0.0107 |

**
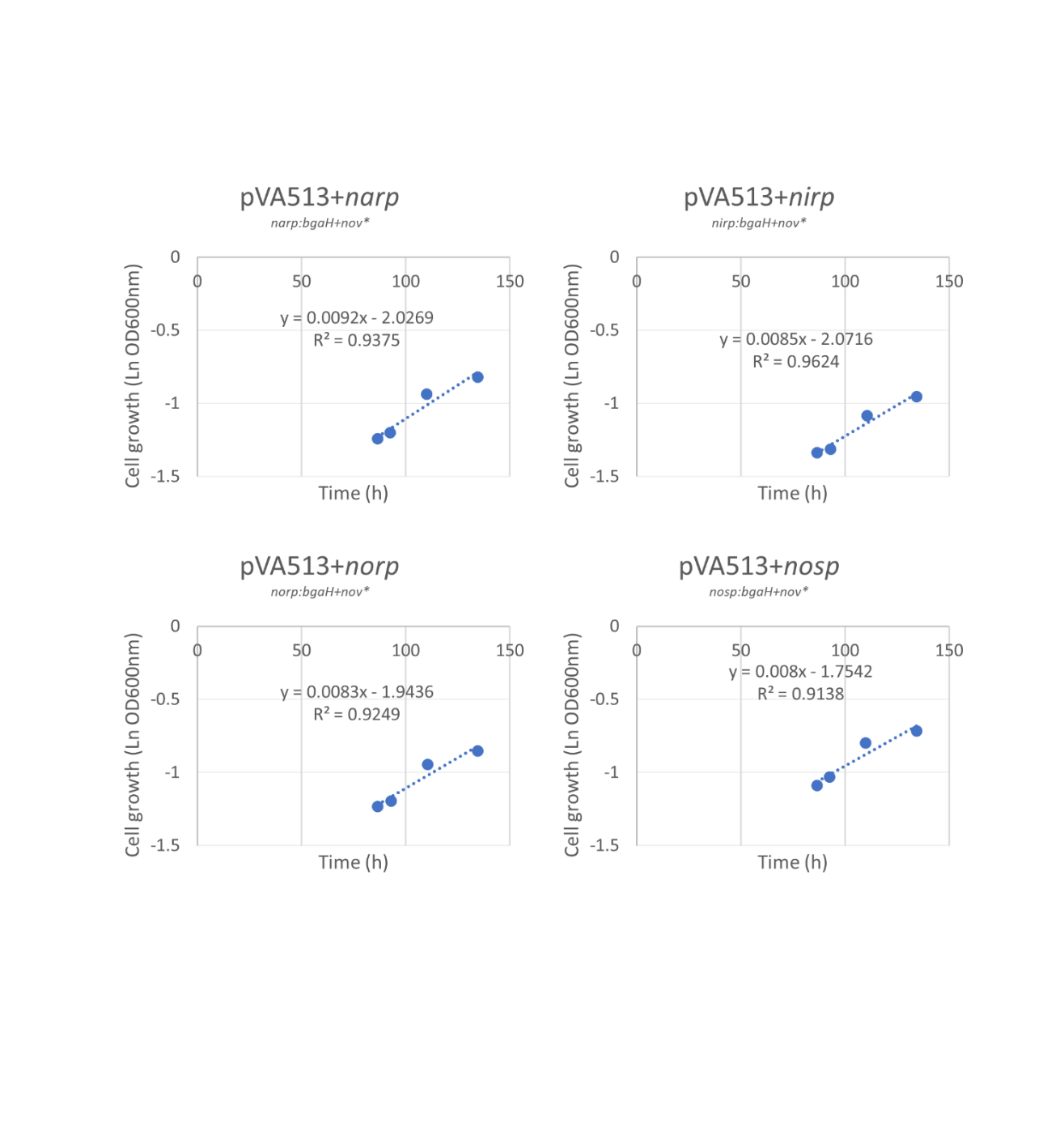
**

**Supplementary Figure 5.** Semi-log plots of the mean of the three replicates for exponential phase cell growth of DMSO-supplemented anaerobic cultures. Titles of each graph represent the specific strain used for that study. pVA513 refers to the plasmid used that carries the reporter gene (*bgaH* gene). *narp* (nitrate reductase promoter), *nirp* (nitrite reductase promoter), *norp* (nitric oxide reductase promoter), and *nosp* (nitrous oxide reductase promoter) refer to the promoter cloned in the plasmid. *nov:* novobiocin resistance.

**
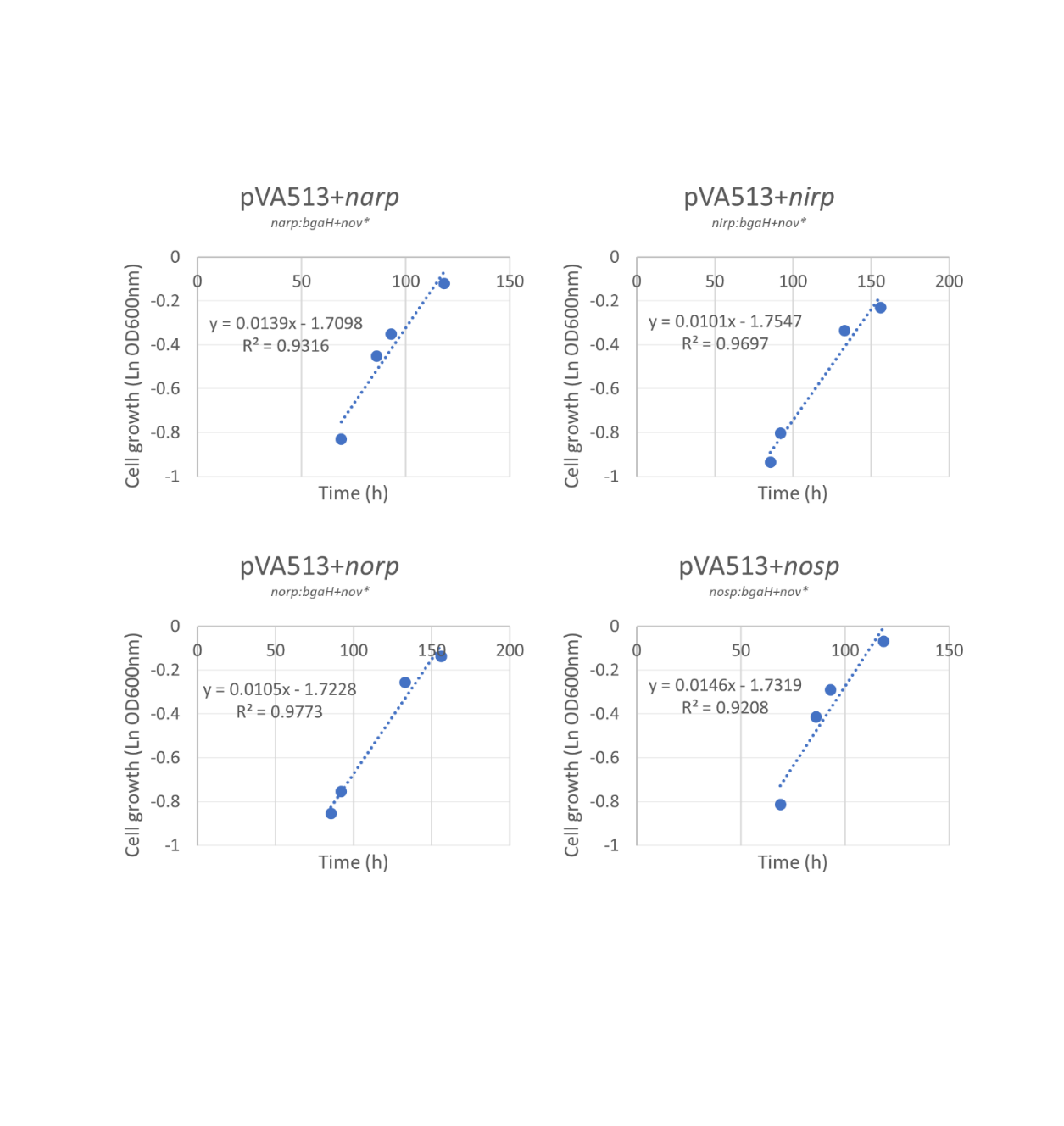
**

**Supplementary Figure 6.** Semi-log plots of the mean of the three replicates for exponential phase cell growth of nitrate-supplemented anaerobic cultures. Titles of each graph represent the specific strain used for that study. pVA513 refers to the plasmid used that carries the reporter gene (*bgaH* gene). *narp* (nitrate reductase promoter), *nirp* (nitrite reductase promoter), *norp* (nitric oxide reductase promoter), and *nosp* (nitrous oxide reductase promoter) refer to the promoter cloned in the plasmid. *nov:* novobiocin resistance.

**
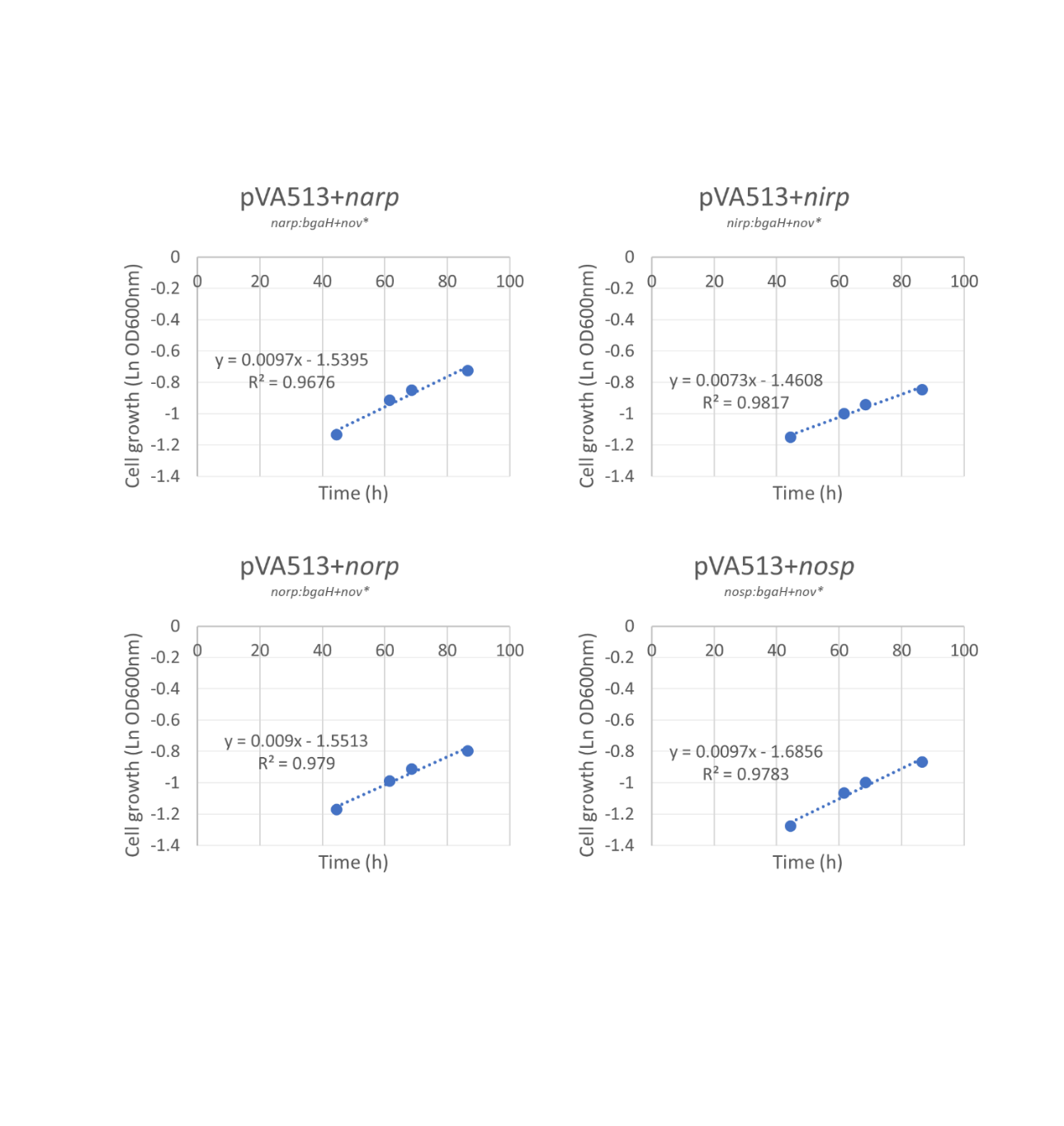
**

**Supplementary Figure 7.** Semi-log plots of the mean of the three replicates for exponential phase cell growth of nitrite-supplemented anaerobic cultures. Titles of each graph represent the specific strain used for that study. pVA513 refers to the plasmid used that carries the reporter gene (*bgaH* gene). *narp* (nitrate reductase promoter), *nirp* (nitrite reductase promoter), *norp* (nitric oxide reductase promoter), and *nosp* (nitrous oxide reductase promoter) refer to the promoter cloned in the plasmid. *nov:* novobiocin resistance.

**Supplementary Table 8.** Promoter regions across the genome that present the same (or very similar) motif found in the promoter regions of the main denitrification genes. *For this bioinformatic analysis the promoter region for each gene was considered as the whole space between two genes. In the case that this space is between two genes transcribed in opposite directions the two genes are listed, being always the closest gene the one that appears in the sixth column. Proteins previously reported as denitrification accessory proteins are in bold.

| Location | start | stop | p-value | motif | Closest gene | Gene product | Closest gene* | Gene product |
| --- | --- | --- | --- | --- | --- | --- | --- | --- |
| Chr | 142203 | 142214 | 5.62E-08 | CGAACATGTTCG | **E6P09_RS00725** | **multicopper oxidase domain-containing protein** | E6P09_RS00730 | helix-turn-helix domain-containing protein |
| Chr | 145214 | 145225 | 5.62E-08 | CGAACATGTTCG | E6P09_RS00745 | CGCGG family rSAM-modified RiPP protein | E6P09_RS00750 | cbb3-type cytochrome c oxidase subunit I (nor) |
| Chr | 149575 | 149586 | 5.62E-08 | CGAACATGTTCG | E6P09_RS00760 | halocyanin domain-containing protein |  |  |
| Chr | 155152 | 155163 | 3.76E-07 | CGAATATGTTCG | **E6P09_RS00790** | **hypothetical protein (pseudogene)** |  |  |
| Chr | 151382 | 151393 | 3.76E-07 | CGAATATGTTCG | E6P09_RS00775 | copper-containing nitrite reductase (*nirK*) |  |  |
| Chr | 150859 | 150870 | 8.22E-07 | CGAATATATTCG | E6P09_RS00770 | DUF2249 domain-containing protein | E6P09_RS00765 | TIGR04053 family radical SAM/SPASM domain-containing protein |
| Chr | 469187 | 469198 | 3.76E-06 | CGAACATTCTCG | E6P09_RS02330 | hypothetical protein |  |  |
| Chr | 1974093 | 1974104 | 4.50E-06 | CGAAGATATTCG | E6P09_RS10145 | FUN14 domain-containing protein | E6P09_RS10150 | GTPase HflX |
| Chr | 1773828 | 1773839 | 7.13E-06 | CGACCATGGTCG | E6P09_RS09025 | ABC transporter substrate-binding protein | E6P09_RS09020 | proline dehydrogenase family protein |
| Chr | 2806870 | 2806881 | 7.13E-06 | CGAACACGGTCG | E6P09_RS19920 | hypothetical protein |  |  |
| Chr | 2306535 | 2306546 | 1.14E-05 | CGAATTTGTTCG | E6P09_RS11805 | group 1 truncated haemoglobin |  |  |
| Chr | 2099405 | 2099416 | 1.14E-05 | CGAATAGGTTCG | E6P09_RS10785 | MOSC domain-containing protein |  |  |
| Chr | 1728140 | 1728151 | 1.49E-05 | CGAACGTTGTCG | E6P09_RS08755 | hypothetical protein | E6P09_RS08760 | NAD-binding protein |
| Chr | 2776335 | 2776346 | 1.49E-05 | CGAACATTGGCG | E6P09_RS14265 | hypothetical protein |  |  |
| Chr | 1284089 | 1284100 | 1.75E-05 | CGAATATTTTGG | E6P09_RS06485 | PadR family transcriptional regulator | E6P09_RS06480 | inorganic diphosphatase |
| Chr | 923948 | 923959 | 2.21E-05 | CGAACATTTATG | E6P09_RS04630 | cell division ATPase MinD | E6P09_RS04625 | peptide chain release factor aRF-1 |
| Chr | 1865511 | 1865522 | 2.21E-05 | CGACTATTTTCG | E6P09_RS09485 | hypothetical protein | E6P09_RS09480 | hypothetical protein |
| Chr | 725159 | 725170 | 2.21E-05 | CGGACATTTTTG | E6P09_RS03515 | DHH family phosphoesterase |  |  |
| Chr | 1607560 | 1607571 | 2.21E-05 | CGAACGTTTTTG | E6P09_RS08120 | phospholipase D-like domain-containing protein |  |  |
| Chr | 1552155 | 1552166 | 2.21E-05 | CGACCATTTTTG | E6P09_RS07865 | ribose 1 5-bisphosphate isomerase |  |  |
| Chr | 2452469 | 2452480 | 2.21E-05 | CGAACGTTTTTG | E6P09_RS12485 | hypothetical protein |  |  |
| Chr | 2636471 | 2636482 | 2.36E-05 | CGAATCTGGTCG | E6P09_RS13485 | hypothetical protein |  |  |
| Chr | 1806771 | 1806782 | 2.36E-05 | CGTACATGGTTG | E6P09_RS09185 | xanthine permease |  |  |
| Chr | 1482231 | 1482242 | 2.72E-05 | CCAATATTTTCG | E6P09_RS07475 | hypothetical protein |  |  |
| Chr | 2174636 | 2174647 | 2.95E-05 | CGCATATGTTTG | E6P09_RS11130 | phosphoribosylformylglycinamidine cyclo-ligase |  |  |
| Chr | 1543291 | 1543302 | 3.03E-05 | CAAATATGTTTG | E6P09_RS07820 | S9 family peptidase | E6P09_RS07815 | hypothetical protein |
| Chr | 2586516 | 2586527 | 3.03E-05 | TGAATATGTTTG | E6P09_RS13240 | substrate-binding domain-containing protein |  |  |
| Chr | 2899621 | 2899632 | 3.46E-05 | CGAATATAGCCG | E6P09_RS14875 | PstS family phosphate ABC transporter substrate-binding protein | E6P09_RS14880 | phosphate uptake regulator PhoU |
| Chr | 101736 | 101747 | 3.46E-05 | CGAGTATTGTCG | E6P09_RS00555 | MoxR family ATPase |  |  |
| Chr | 353992 | 354003 | 3.78E-05 | AGAATATAGTCG | E6P09_RS01680 | MFS transporter |  |  |
| Chr | 232377 | 232388 | 3.78E-05 | TGAATATAGTCG | E6P09_RS01090 | PRC-barrel domain-containing protein | E6P09_RS01085 | hypothetical protein |
| Chr | 224358 | 224369 | 3.78E-05 | CGAATATAGTCT | E6P09_RS01060 | hypothetical protein | E6P09_RS01065 | SDR family oxidoreductase |
| Chr | 2115447 | 2115458 | 4.05E-05 | CGAATATTTCTG | E6P09_RS10850 | fructose PTS transporter subunit IIC |  |  |
| Chr | 143508 | 143519 | 4.05E-05 | CGAATGTTTTTG | E6P09_RS00735 | helix-turn-helix domain-containing protein | **E6P09_RS00740** | **halocyanin domain-containing protein** |
| Chr | 2081087 | 2081098 | 4.05E-05 | CGAATCTTTTTG | E6P09_RS10685 | 1 4-dihydroxy-2-naphthoyl-CoA synthase | E6P09_RS10690 | CopD family protein |
| Chr | 856614 | 856625 | 4.14E-05 | CGAATATGGGTG | E6P09_RS04260 | transcription initiation factor IIB family protein | E6P09_RS04265 | hypothetical protein |
| Chr | 1189780 | 1189791 | 4.35E-05 | CGAATATATTTC | E6P09_RS06000 | hypothetical protein |  |  |
| Chr | 267340 | 267351 | 4.65E-05 | CGAATGTAGTTG | E6P09_RS01265 | hypothetical protein |  |  |
| Chr | 213147 | 213158 | 4.92E-05 | AGAATATAGTTG | E6P09_RS01030 | surface glycoprotein |  |  |
| Chr | 2209451 | 2209462 | 4.92E-05 | CAAATATTGTTG | E6P09_RS11280 | molecular chaperone DnaK |  |  |
| Chr | 28677 | 28688 | 4.92E-05 | CGAATATAGTTC | E6P09_RS00160 | VOC family protein |  |  |
| Chr | 153713 | 153724 | 6.02E-05 | CGAACCGGTTCG | E6P09_RS00785 | cupin domain-containing protein |  |  |
| Chr | 976485 | 976496 | 6.02E-05 | CGAACCGGTTCG | E6P09_RS04890 | hypothetical protein |  |  |
| Chr | 1688624 | 1688635 | 6.02E-05 | CGAACAAGTACG | E6P09_RS08540 | PGF-CTERM sorting domain-containing protein |  |  |
| Chr | 152650 | 152661 | 7.04E-05 | CGGACATGTTCA | E6P09_RS00780 | hypothetical protein |  |  |
| Chr | 1527667 | 1527678 | 7.04E-05 | CGAACGTGTTCC | E6P09_RS07725 | pyridoxamine 5\\'-phosphate oxidase family protein | E6P09_RS07720 | cation-transporting P-type ATPase |
| Chr | 621219 | 621230 | 7.32E-05 | CGAACCTCGTCG | E6P09_RS03040 | ABC transporter ATP-binding protein |  |  |
| Chr | 1065215 | 1065226 | 7.79E-05 | CGAGCATACTCG | E6P09_RS05390 | hypothetical protein | E6P09_RS05395 | hypothetical protein |
| Chr | 2655545 | 2655556 | 7.79E-05 | CGAACACTCTCG | E6P09_RS19755 | hypothetical protein |  |  |
| Chr | 231656 | 231667 | 7.79E-05 | CGAACACTATCG | E6P09_RS01085 | hypothetical protein | E6P09_RS01090 | PRC-barrel domain-containing protein |
| Chr | 1468692 | 1468703 | 7.79E-05 | CGAACACACTCG | E6P09_RS07400 | cox cluster protein |  |  |
| Chr | 2886061 | 2886072 | 7.83E-05 | TGAACATCGTCG | E6P09_RS14825 | CBS domain-containing protein | E6P09_RS14815 | hypothetical protein |
| Chr | 634643 | 634654 | 8.94E-05 | CGATGATTTTCG | E6P09_RS03100 | ORC1-type DNA replication protein | E6P09_RS03095 | Era-like GTP-binding protein |
| Chr | 1413849 | 1413860 | 8.94E-05 | CGAACTTATTAG | E6P09_RS07160 | DNA polymerase | E6P09_RS07155 | hypothetical protein |
| Chr | 2775138 | 2775149 | 9.19E-05 | AGAACATTATCG | E6P09_RS14265 | hypothetical protein |  |  |
| Chr | 600145 | 600156 | 9.19E-05 | CAAACATTCTCG | E6P09_RS02955 | hypothetical protein |  |  |
| Chr | 1028234 | 1028245 | 9.46E-05 | CGAACATGGGGG | E6P09_RS05170 | hypothetical protein |  |  |
| Chr | 1926042 | 1926053 | 9.46E-05 | CGAACCTGGTAG | E6P09_RS09875 | Lrp/AsnC family transcriptional regulator |  |  |
| Chr | 2058173 | 2058184 | 9.46E-05 | CGAAAAAGGTCG | E6P09_RS10575 | ABC transporter ATP-binding protein |  |  |
| Chr | 1592456 | 1592467 | 9.46E-05 | CGAAGACGGTCG | E6P09_RS08040 | hypothetical protein |  |  |
| pHME132 | 75045 | 75056 | 2.72E-05 | CAAATATATTCG | E6P09_RS19145 | alpha/beta hydrolase |  |  |
| pHME132 | 123916 | 123927 | 3.78E-05 | GGAACATAGTTG | E6P09_RS19415 | MarR family transcriptional regulator |  |  |
| pHME132 | 122621 | 122632 | 6.02E-05 | CGAACCGGTTCG | E6P09_RS19905 | hypothetical protein |  |  |
| pHME322 | 4426 | 4437 | 5.62E-08 | CGAACATGTTCG | E6P09_RS17385 | plastocyanin/azurin family copper-binding protein (*nos* operon) | E6P09_RS17390 | hypothetical protein |
| pHME322 | 20146 | 20157 | 1.73E-06 | CGAATATTGTTG | E6P09_RS17470 | TetR/AcrR family transcriptional regulator | E6P09_RS17465 | hypothetical protein (*nar* operon) |
| pHME322 | 4588 | 4599 | 2.14E-06 | CGAAGATGTTCG | E6P09_RS17390 | hypothetical protein | E6P09_RS17385 | plastocyanin/azurin family copper-binding protein (*nos* operon) |
| pHME322 | 6870 | 6881 | 2.88E-06 | CGAACACGTTCG | E6P09_RS17400 | hypothetical protein |  |  |
| pHME322 | 192906 | 192917 | 3.46E-06 | AGAACATGTTCG | E6P09_RS18190 | ABC transporter substrate-binding protein | E6P09_RS18185 | hypothetical protein |
| pHME322 | 201652 | 201663 | 4.71E-06 | CGAAGATGGTCG | E6P09_RS18225 | ABC transporter permease subunit | E6P09_RS18220 | hypothetical protein |
| pHME322 | 317695 | 317706 | 4.14E-05 | CGAATGTGGTTG | E6P09_RS18735 | ABC transporter ATP-binding protein | E6P09_RS18740 | hypothetical protein |
| pHME322 | 63378 | 63389 | 6.02E-05 | CGAACCCGTTCG | E6P09_RS17615 | cupin domain-containing protein |  |  |
| pHME322 | 131418 | 131429 | 8.94E-05 | CGACCATCTTTG | E6P09_RS17925 | NAD(P)-dependent alcohol dehydrogenase | E6P09_RS17920 | alpha/beta hydrolase |
| pHME505 | 437936 | 437947 | 3.51E-06 | CGAACATCGTCG | E6P09_RS17090 | DegT/DnrJ/EryC1/StrS family aminotransferase | E6P09_RS17085 | hypothetical protein |
| pHME505 | 435833 | 435844 | 1.49E-05 | CGAACACTGTCG | E6P09_RS17080 | MarR family transcriptional regulator |  |  |
| pHME505 | 46479 | 46490 | 1.77E-05 | CGAATATGGTAG | E6P09_RS15300 | hypothetical protein | E6P09_RS15295 | ArsA family ATPase |
| pHME505 | 167496 | 167507 | 4.14E-05 | CGTATATGGTTG | E6P09_RS19800 | hypothetical protein |  |  |
| pHME505 | 68603 | 68614 | 4.14E-05 | CGAATACGGTTG | E6P09_RS15400 | NAD-binding protein | E6P09_RS15395 | thiamine pyrophosphate-binding protein |
| pHME505 | 455721 | 455732 | 7.79E-05 | CGAACACTATCG | E6P09_RS17160 | hypothetical protein | E6P09_RS17155 | DUF1616 domain-containing protein |
| pHME505 | 13418 | 13429 | 7.79E-05 | CGAACATACACG | E6P09_RS15175 | hypothetical protein |  |  |
| pHME505 | 400264 | 400275 | 7.83E-05 | CAAACATCGTCG | E6P09_RS16935 | sugar kinase | E6P09_RS16930 | bifunctional 4-hydroxy-2-oxoglutarate aldolase/2-dehydro-3-deoxy-phosphogluconate aldolase |
| pHME505 | 155510 | 155521 | 8.94E-05 | CGAACATATGAG | E6P09_RS15760 | helix-turn-helix transcriptional regulator | E6P09_RS15765 | hypothetical protein |
| pHME505 | 334295 | 334306 | 9.46E-05 | CGGACATGGTGG | E6P09_RS16660 | orc1/cdc6 family replication initiation protein |  |  |
